# Advancing Forensic Human Chronological Age Estimation: Biochemical, Genetic, and Epigenetic Approaches from the Last 15 Years: A Systematic Review

**DOI:** 10.3390/ijms26073158

**Published:** 2025-03-28

**Authors:** Beatrice Marcante, Laura Marino, Narjis Elisa Cattaneo, Arianna Delicati, Pamela Tozzo, Luciana Caenazzo

**Affiliations:** 1 Legal Medicine Unit, Department of Cardiac, Thoracic, Vascular Sciences and Public Health, University of Padova, 35122 Padova, Italy; beatrice.marcante@phd.unipd.it (B.M.); laura.marino@studenti.unipd.it (L.M.); narjiselisa.cattaneo@studenti.unipd.it (N.E.C.); arianna.delicati@phd.unipd.it (A.D.); pamela.tozzo@unipd.it (P.T.); 2 Unit of Biostatistics, Epidemiology and Public Health, Department of Cardiac, Thoracic, Vascular Sciences and Public Health, University of Padova, 35121 Padova, Italy

**Keywords:** age estimation, epigenetics, forensic genetics, review

## Abstract

Forensic age estimation is crucial for identifying unknown individuals and narrowing suspect pools in criminal investigations. Over the past 15 years, significant progress has been made in using biochemical, genetic, and epigenetic markers to estimate chronological age. Methods: From research on PubMed a total of 155 studies, related to advancements in age prediction techniques, were selected following PRISMA guidelines. Studies considered eligible dealt with radiocarbon dating, aspartic acid racemization, mitochondrial DNA analysis, signal joint T-cell receptor excision circles, RNA analysis, telomeres, and DNA methylation in the last 15 years and were summarized in a table. Results: Despite these advancements, challenges persist, including variability in prediction accuracy, sample degradation, and the lack of standardization and reproducibility. DNA methylation emerged as the most promising approach capable of high accuracy across diverse populations and age ranges. Multimodal methods integrating several biomarkers show promise in improving reliability and addressing these limitations. Conclusion: While significant progress has been made, further standardization, validation, and technological integration are needed to enhance forensic age estimation. These efforts are essential for meeting the growing demands of forensic science while addressing ethical and legal considerations.

## 1. Introduction

The estimation of age at death can provide important information in forensic science. Over the years, there have been different attempts in the forensic community to establish and validate methods to address this issue. The natural process of aging triggers progressive changes in tissues and organs at the molecular level throughout a person’s lifetime. Traditionally, forensic scientists have had limited options for estimating the age of individuals in practical casework. Techniques such as morphological examination of bones or amino acid racemization analysis of teeth are available but are constrained by their reliance on specific types of samples. Moreover, these techniques are not applicable to biological fluids, which are more frequently encountered at crime scenes. New methods lend themselves to improving estimation of the age of a person either dead or alive. Furthermore, the ability to predict a subject’s age could find significant applications in narrowing down the pool of suspects when estimating the age of an individual who left a trace at a crime scene. The capability to estimate the chronological age of an unidentified individual from a DNA sample can offer invaluable insights to investigators. This becomes even more powerful when combined with the prediction of externally visible characteristics (EVCs), many of which are influenced by age.

An essential aspect to consider is the difference between chronological age and biological age. Chronological age refers to the amount of time that has passed since birth, while biological age reflects the actual physiological condition of the body at a cellular and molecular level. Biological age is shaped by various factors, including chronic illnesses, epigenetic changes, lifestyle choices, and environmental influences [1].

Humans’ exceptional longevity represents an extreme phenotype and studies have shown that centenarians exhibit a remarkable compression of morbidity and a resistance to otherwise lethal illnesses occurring earlier in life, providing important insights into the underlying molecular mechanisms of aging [2]. In general, aging is the gradual decline of physiological functions leading to age-dependent fitness loss, diseases, and eventually mortality [3].

The need for accurate techniques to estimate human biological age in forensic contexts has never been as important as it has been in recent years. This is due to several factors, including the increasing number of unidentified cadavers and human remains, cases requiring age estimation in living individuals without valid proof of date of birth, and the wide necessity of estimating an unknown stain donor’s age [4].

There have been different approaches for estimating age at death in corpses as well as in living persons, but a common challenge remains the lack of standardized methods and sampling procedures, along with concerns about the accuracy of these methods [5]. To address these limitations, forensic experts have started incorporating genetic knowledge into their analyses, utilizing biomarkers such as signal joint T-cell receptor excision circles, telomere length, and somatic gene rearrangements. However, these genetic markers often demonstrate limited accuracy or are highly susceptible to DNA degradation, especially in samples recovered from crime scenes. Recently, epigenetics has emerged as a promising tool for forensic age prediction [6]. Cytosine methylation at CpG sites, in particular, has been extensively studied and is now recognized as a promising epigenetic marker for determining chronological age [7]. Several researchers have pinpointed CpG sites where methylation levels show a strong correlation with age. Leveraging this knowledge, they have developed age prediction models focusing on a limited number of these sites, enabling accurate age estimation. These models can be integrated into various assays using advanced technologies such as pyrosequencing, single-base extension (SBE) with SNaPshot chemistry, MassARRAY, methylation-specific PCR (MSP), methylation-sensitive high-resolution melting (MS-HRM), next-generation sequencing (NGS), and a range of other methodologies. These approaches have been widely employed in recent years, leading to a flourishing body of scientific literature compared to all other methods, with varying degrees of success.

Establishing the age in cadavers, human remains, or living people may clarify issues with significant legal, ethical, and social implications for individuals as well as for the community [8]. Ideally, an age prediction system suitable for forensic purposes should exhibit specific properties, including applicability to different tissues, reproducibility across diverse populations and across various technology platforms, and coverage of the entire age spectrum. The validation of these systems among different research groups will be critical for driving future advancements.

Furthermore, while most forensic age predictors have been previously developed using whole blood samples, recent studies have also investigated other tissues, such as saliva or different tissues from postmortem samples [9].

However, we have to underline that, independently from the techniques used, the low quality and quantity of DNA usually recovered from crime scene stains are crucial and currently represent the limiting factor not only in the progress of “traditional” forensic genetics but also for the application of age prediction epigenetic techniques, independently from the biological sample type tested.

In such a framework, methods for age estimation have to fulfil specific demands. The choice of a method has to consider the specific circumstances of the individual case, which involves assessing the accuracy required by the case, which age range is to be expected, which epigenetic effects have to be considered, and, for example, in cadavers the type of biological material analyzed and in living individuals the ethical and legal regulations considered.

Furthermore, specialized and trained laboratories and personnel are required for the application of all effective methods in order to avoid systematic errors [10]. Notably, while numerous methods have been applied by different research groups, each of them has applied its own methodological protocol and analytical workflow. This led to severe limitations in comparability, reproducibility, and verification of results. In a time in which quality control has achieved great importance in all fields of biomedical sciences it is surprising how few attempts have been made to find common standardization, calibration, and evaluation procedures for methods for human age prediction. At the moment, there are no generally accepted guidelines concerning quality assurance in human chronological age prediction. Efforts in these directions are necessary in order to guarantee quality standards and adequate answers to the important legal, ethical, and social issues of age estimation in forensic medicine [4].

Within this context, this systematic review aims to explore recent advancements in the application of the most commonly used methods for predicting human chronological age in forensic science. It focuses on research published over the past 15 years, with the goal of critically evaluating the strengths and limitations of each technique. The review also addresses challenges related to the adaptability of these methods in forensic laboratories, the precision required to achieve narrow age ranges, the reproducibility both within the same laboratory and across different laboratories, and, importantly, the development of effective prediction tools.

## 2. Materials and Methods

**Figure 1:**
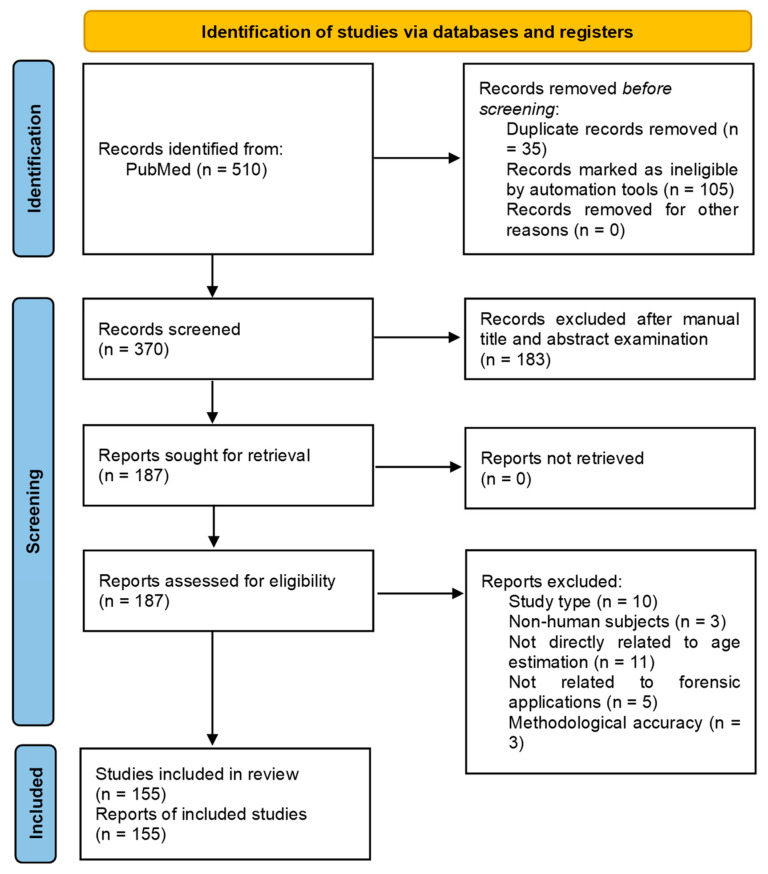
Preferred Reporting Items for Systematic Reviews and Meta-Analyses (PRISMA) 2020 flow diagram.

This review was performed in adherence to the Preferred Reporting Items for Systematic Reviews and Meta-Analyses (PRISMA) guidelines and checklist without a dedicated review protocol and it was not registered [11]. A systematic literature review regarding different methods for human age prediction was conducted using the public electronic database PubMed. The works were selected according to the following queries: (Carbon 14) AND ((age estimation) OR (age prediction)) AND (forensic); (racemization of aspartic acid) AND ((age estimation) OR (age prediction)) AND (forensic); (T-cell receptor) AND ((age estimation) OR (age prediction)) AND (forensic); (mtDNA) AND ((age estimation) OR (age prediction)) AND (forensic); (RNA) AND ((age estimation) OR (age prediction)) AND (forensic); (Telomere) AND ((age estimation) OR (age prediction)) AND (forensic); and (DNA Methylation) AND ((age estimation) OR (age prediction)) AND (forensic). A total of 510 works were identified through database searching. A total of 35 duplicate records were removed. Subsequently, different inclusion criteria were considered and 105 records were removed. Inclusion criteria included: English language restrictions, full-text availability, and a predefined time interval limit of the last 15 years (from 2010 to 2024, extremes included). Then, the process continued through the screening of titles and abstracts of the remaining 370 records. In this phase, 183 records were excluded based on the following criteria: the type of study (reviews, meta-analyses, case reports, and comments were not included), focused on non-human subjects, not directly related to age estimation, not related to forensic applications, or the methodological process was not accurately described. The article search was conducted by four independent researchers. No automation tools were used for the full-text data analysis. In cases of any doubt, the consensus opinions of the two research supervisors were solicited. Both research supervisors worked independently. A total of 187 articles were further evaluated by full-text examination to exclude irrelevant content based always on the previously criteria. After a full-text reading of the selected papers, 155 were considered eligible and included in the review. The most relevant information was collected and organized in two tables to give a clear summary of the study’s results (Supplementary Materials Tables S1 and S2). The PRISMA flow chart in Figure 1 summarizes the workflow of the screening and selection process described above. This systematic review analyzes in detail different studies, which deal with different approaches and methods to human chronological age estimation. In order to provide the reader with a better understanding, we grouped the selected studies on the basis of the methods applied: (a) radiocarbon dating; (b) aspartic acid racemization (AAR); (c) mitochondrial DNA (mtDNA) analysis; (d) signal joint T-cell receptor excision circles (sjTRECs); (e) RNA analysis; (f) telomeres; (g) DNA methylation. Moreover, data were divided considering different biological material sources and individual characteristics such as gender, age, and vital status.

## 3. Results

In molecular biology, most age estimation techniques rely on degenerative changes associated with the aging process, which are intrinsically subject to individual variability in their timing. Consequently, in forensic science, age estimation methods based on biochemical processes—such as aspartic acid racemization (AAR) and carbon-14 quantification—have gained prominence, as they involve measurable chemical changes that occur universally and predictably over time in specific compounds, offering greater reliability.

### 3.1. Radiocarbon Dating

The application of radiocarbon (14C) dating in forensic age estimation is based on the distinct carbon signature created by nuclear bomb tests conducted between 1955 and 1963, which led to a significant increase in atmospheric 14C levels. This radioactive carbon was absorbed by living organisms, including humans, as they formed biological tissues. During tooth development, the 14C incorporated into the enamel remains fixed, preserving the radiocarbon signature throughout an individual’s life. As a result, by measuring the 14C concentration in enamel, researchers can estimate the individual’s birth year with high precision, as the levels of 14C in the enamel correspond to atmospheric levels at the time of tooth formation. Over time, atmospheric 14C concentrations have decreased due to mixing with large marine and terrestrial carbon reservoirs [12]. To estimate age, the measured 14C in tooth enamel is compared to a calibration curve representing atmospheric 14C levels, which peaked during the 1955–1963 bomb pulse and subsequently declined [13,14].

Accelerator mass spectrometry (AMS) is the most commonly used and precise technique for measuring isotope ratios. Kondo-Nakamura et al. [15] analyzed eight teeth from five Japanese individuals (five males and one female aged between 29 and 75 years), recommending that accuracy in age estimation can be improved by analyzing multiple teeth from the same individual or different regions of a single tooth, as these correspond to various stages of enamel formation. This approach increases precision by accounting for the distinct growth rates of different teeth and their respective developmental stages, achieving a difference between actual and predicted age of birth of approximately ± 2 years. Alkass et al. [16] examined 95 teeth from 84 individuals across different geographic regions, finding that teeth formed before 1955 exhibited negative Δ14C values, while those formed during the bomb pulse (1955–1963) had an average birth year error of 1.9 ± 1.4 years. Teeth formed after the peak (post-1963) showed even better accuracy, with an error of 1.3 ± 1.0 years. Furthermore, no significant geographic variation was observed in the average birth year estimation error. Teglind et al. [17] combined 14C and amino acid racemization (AAR) analyses on 63 teeth and 24 bones from 52 individuals. Among the 34 cases, 18 cases were found to be postbomb, indicating tooth crown formation after 1955, and 9 of these were identified. In these individuals, a high correlation between the estimated and true year of birth was found with an average absolute error of 1.18 ± 0.83 years. Notably, 14C analysis of tooth enamel and dentin demonstrated excellent precision, even in samples exposed to environmental elements such as soil, air, and water for extended periods, ruling out significant contamination. In contrast to enamel and dentin, bone tissue undergoes continuous remodeling, leading to carbon turnover at a rate that complicates the use of 14C analysis for dating birth. Alkass et al. [18] demonstrated the combined use of 14C and AAR in 44 teeth from 41 Swedish individuals, revealing a good correlation between the methods (R^2^ = 0.66, *p* < 0.05). Radiocarbon analysis achieved an overall absolute error of 1.0 ± 0.6 years, while aspartic acid racemization exhibited precision with an error of 5.4 ± 4.2 years. While radiocarbon analysis provides an estimated year of birth, racemization analysis determines the chronological age of the individual at the time of death. Moreover, four additional teeth of an unsolved homicide case were analyzed with radiocarbon and aspartic acid racemization, indicating that the victim was probably born around 1942 and died around 1988.

These studies highlight the advantages of using AMS for radiocarbon dating in forensic contexts, offering high precision and the ability to analyze small samples without damaging the specimen. However, there are notable limitations, including the high cost and labor-intensive sample preparation process. Additionally, organic materials that are poorly preserved or contaminated may yield inaccurate results. The accuracy of the method also diminishes for individuals born before 1955, when atmospheric 14C levels were significantly lower. Therefore, while radiocarbon dating offers a highly accurate means of estimating birth years, particularly for individuals born during the period of increased 14C, its application in forensic contexts is subject to certain limitations, including cost, sample quality, and diminishing precision for those born before 1955.

### 3.2. Aspartic Acid Racemization (AAR)

Racemization is a chemical process in which the natural L-form (levorotatory) of an amino acid is gradually converted into its mirror-image D-form (dextrorotatory). Aspartic acid (Asp) is particularly prone to racemization, especially when incorporated into proteins, due to the chemical instability of the peptide bond. This process is influenced by conditions such as elevated temperatures, extreme pH, oxidative stress, and the structural properties of the protein [19]. Over time, D-aspartic acid residues accumulate in a predictable, time-dependent manner, making racemization a valuable marker for estimating the age of biological samples [20].

Stable, long-lived proteins, such as dentin in teeth or elastin in cartilage, are particularly suitable for age estimation, as they accumulate measurable levels of D-aspartic acid over time. By determining the ratio of D- to L-aspartic acid and comparing it to established racemization rates [21], researchers can estimate the sample’s chronological age. However, factors like temperature, humidity, protein composition, and exposure to reactive oxygen species can affect the racemization rate, necessitating careful calibration.

High-performance liquid chromatography (HPLC), often combined with mass spectrometry (MS), is the standard method for quantifying D-aspartic acid. HPLC separates chemical components in protein extracts, while MS confirms the identity and quantity of D-aspartic acid with high sensitivity and precision.

Griffin et al. [22] conducted a study to evaluate the natural variability in aspartic acid racemization (AAR) ratios across different types of human tooth enamel (*n* = 129), collected from both living individuals and archaeological remains. Their analysis considered the influence of tooth type and its development age on racemization rates. The findings revealed no significant relationship between tooth type and racemization rates after accounting for the developmental age of each tooth. Despite this, significant variability in racemization values was observed between different teeth from the same individual, potentially leading to age estimation discrepancies of up to 25 years, observed in archaeological remains. This variability was attributed to factors such as enamel protein composition, the quantity of enamel sampled, and postmortem environmental conditions, rather than tooth type itself. A different result was observed considering modern individuals for which the minimal paired age estimate was 7.1 years.

In a first study conducted by Sakuma and colleagues [23] both dentin and whole teeth from 12 pairs of canines were analyzed to assess AAR’s correlation with chronological age. They found a strong correlation for dentin samples (r = 0.98) and a slightly lower correlation for whole teeth (r = 0.93). The study highlighted the advantage of using whole teeth, which preserves the sample for additional forensic analyses, such as DNA extraction, while reducing variability from localized sampling in dentin. In addition, they evaluated their model using teeth from two different cases, the result overestimating the age of individuals by approximately 0.12 and 6.86 years, respectively. In a following study, Sakuma et al. [24] analyzed three teeth in correlation with five control teeth which generated a regression line characterized by a strong correlation coefficient (r = 0.99). In this context, the racemization rate of the two groups was not significantly different, with differences in age between −1 and + 3 years when considering pink teeth.

Sirin et al. [25] emphasized the importance of special handling of teeth affected by caries, as caries significantly compromises the AAR process. To address this issue, the study analyzed 99 standardized root dentine samples prepared from 25 wisdom teeth with carious lesions, extracted by dentists due to carious or periodontal diseases, and, as a control, 3 sound wisdom teeth. In control samples they obtained a deviation between estimated and real ages ranging from −1.87 to 3.69 years. This deviation increased significantly in affected samples. Therefore, the researchers concluded by recommending the use of two teeth from the same individual, given the low likelihood of identical caries-induced degradation kinetics in separate teeth.

Lastly, Arany et al. [26] extended the application of racemization to amino acids beyond aspartic acid, including glutamate and alanine. The analysis of 24 premolars collected from patients under periodontal or orthodontics treatment demonstrated that the D/L-aspartic acid ratio provided the most accurate results.

The applicability of AAR has been validated across diverse populations. For example, Wochna et al. [27] applied AAR analysis to root dentin from 75 teeth in a Polish population, including central and lateral incisors, canines, and first premolars. Their study reported high correlation coefficients (r = 0.96–0.98) and standard errors (SEs) of ±2.95 to ±4.84 years, with tooth-specific calibration further enhancing accuracy. Similarly, Rastogi et al. [28] conducted a pilot study using gas chromatography on tooth biopsy specimens from 90 Indian individuals. Despite differences in methodology, the study reported error margins of 0 to + 4 years, comparable to those using HPLC. Chen et al. [29] focused on third-molar dentin in a Chaoshan population (*n* = 58), emphasizing the importance of population-specific calibration. The findings confirmed AAR’s effectiveness and precision in age estimation, with a correlation coefficient of about 0.97 and a mean absolute error (MAE) of 2.19 years, with a standard deviation of ±1.53 years.

Supporting these results, Zapico et al. [30] examined D/L ratios in dentin from 20 third molars in a Spanish cohort, demonstrating a close linear correlation with chronological age (r = 0.91) with an MAE of approximately 5 years in the training set and a difference in accuracy of ±2–6 years in the test set. In another study, Elfawal et al. [31] analyzed 89 upper first premolars from a Kuwaiti population, achieving close correlations (r = 0.97) and a minimal SE of ±1.26 years in a test group, with even lower errors of ±1.12 years in a validation group.

To overcome the challenges posed by limited sample availability, Minegishi et al. [32] developed a heat-treatment approach to accelerate racemization. By heating dentin samples (*n* = 39), they created calibration standards that mimicked the natural aging process, shortening experimental timeframes and improving reproducibility. In particular, through a more detailed analysis of some samples, they demonstrated that AAR can increase significantly if a temperature of 110 °C is considered, showing a strong correlation (r > 0.91) with the relative age of the samples. Moreover, the method obtained an error of ±5 years when age was estimated.

Beyond dentin, alternative tissues such as epiglottis cartilage and sclera have also shown promise for AAR-based age estimation. Matzenauer et al. [33] analyzed AAR on 65 cartilage samples from the epiglottis and 45 purified elastin samples, showing a closer relationship between AAR and age at death in purified elastin from epiglottis (r = 0.84) compared to unprocessed cartilage (r = 0.76). Klumb et al. [34] analyzed 75 scleral tissue samples, observing an increase in the extent of AAR with age. However, the relationship between age and racemization extent (r = 0.92) was lower than in dentin. This is probably due to molecular inhomogeneities of total tissue samples which, therefore, resulted in deviations between ±6.5 and ±32.35 years in the validation set.

In conclusion, AAR-based methods for age estimation demonstrate accuracy and strong correlations with age across various studies. However, practical application is limited by factors such as the destructive nature of analysis, the high sensitivity to postmortem and storage conditions, and the need for population-specific calibration models.

### 3.3. Mitochondrial DNA (mtDNA) Analysis

Mitochondria, double-membrane organelles responsible for producing ATP through oxidative phosphorylation, also generate reactive oxygen species (ROS) as by-products of this process. These ROS, including superoxide and hydrogen peroxide, cause oxidative damage to cellular components, contributing to disease, aging, and senescence [35,36]. Mitochondrial DNA (mtDNA) is particularly prone to ROS-induced damage due to its proximity to the electron transport chain and lack of protective histones, leading to mutagenesis. Somatic mtDNA mutations impair respiratory chain function, increasing ROS production and creating a self-perpetuating cycle of damage. This process leads to the progressive accumulation of mtDNA mutations over time, especially in high-energy, postmitotic tissues such as skeletal muscles, the brain, and the heart [37].

Studies have demonstrated a strong correlation between mtDNA mutations, deletions, or duplications with aging, suggesting their potential use as biomarkers for estimating human chronological age in forensic applications.

For example, Zapico et al. [38] analyzed teeth samples, separating dentin from pulp, from 25 third molars in Catalonia and 24 in Asturias, collected from individuals aged 20–70 years. Instead of targeting specific mutations, they quantified HV2 region amplification in mtDNA using RT-PCR. Since HV2 amplification is influenced by oxidative damage to nucleotides, the study found a negative correlation between defective amplification events and age in dentin from both populations with a significance value ≤ 0.01. However, when analyzing the pulp samples, the correlation between HV2 amplification and age was weaker and not statistically significant.

Similarly, Lacan et al. [39] investigated three types of miniduplications in the mtDNA D-loop (150 bp, 190 bp, and 260 bp) in muscle and bone samples from 82 individuals aged 2–87 years. Using a pair of primers (L336/H335) previously linked to age-related somatic mutations in muscle, the researchers developed a sensitive method combining back-to-back primers with capillary electrophoresis to detect low levels of heteroplasmy. Their findings confirmed a correlation between age and the frequency of duplications, with a higher prevalence observed in muscle tissue compared to bone, with up to three duplications in the first case and two in the second one, predominantly in elder individuals.

### 3.4. Quantification of Signal Joint T-Cell Receptor Excision Circles (sjTRECs)

In molecular biology, one of the most encouraging methods for age estimation is the quantification of signal joint T-cell receptor excision circles (sjTRECs) from blood samples. T-cell receptor excision circles (TRECs) are extrachromosomal DNA by-products generated during the rearrangement of gene segments encoding the variable regions of the TCR α- and β-chains in developing TCR αβ+ lymphocytes. These episomal DNA fragments are stable and non-replicative, meaning they are exclusively present in newly emigrated (naïve) T-cells from the thymus; as thymic output diminishes with age due to thymic involution [40], the number of TRECs declines. Among these, sjTRECs—produced specifically during the rearrangement of the TCR α-chain gene—are widely recognized as a reliable surrogate marker of thymic activity [41]. Research has explored the potential of sjTRECs quantification for age estimation, employing qPCR to analyze blood samples from individuals of known ages. Studies conducted across various populations, such as Chinese, Korean, Egyptian, Dutch, and Japanese groups, have consistently demonstrated a strong negative correlation between sjTRECs levels and age. For instance, a study by Ou et al. [42] in a Chinese cohort (*n* = 248) reported a correlation coefficient of approximately −0.82 and an error margin of approximately ±10.47 years. Similarly, Cho et al. [43] refined the method using TaqMan probes in a Korean population (*n* = 172), achieving an error margin of ±8.49 years. Comparable findings have been reported in other studies: Ibrahim et al. [44] analyzed blood samples from 153 healthy Egyptian individuals, obtaining an SE of ± 7.35 years, Zubakov et al. [45] obtained an error of 8.9 years from 195 healthy Dutch individuals, and, lastly, Yamanoi et al. [46] reported an error of ±8.0 years when considering the 194 Japanese samples for which the analysis was successfully performed. Moreover, the latter observed a significant decrease in sjTRECs quantification when bloodstains were stored for over 1 year. To expand the applicability of sjTRECs quantification, some studies assessed its use in dried bloodstains, which are more commonly encountered in forensic scenarios. Ou et al. [47] analyzed bloodstains from 264 Chinese individuals stored under various conditions and found a strong negative correlation between sjTREC levels and donor age (r = −0.871) with an SE of ±9.42 years. However, when further samples stored for different periods were analyzed, they observed that older bloodstains exhibited reduced accuracy due to time-dependent degradation of sjTREC levels, emphasizing the need for standardized methodologies to account for storage effects.

The relationship between sjTRECs and chronological age can also be influenced by health conditions. The immune systems of individuals affected by certain diseases may show signs of early immune senescence, weakening the correlation between sjTREC levels and chronological age. For instance, Cho et al. [48] examined 149 blood samples from a biobank of individuals with adverse drug reaction, including anaphylaxis, urticaria, drug reaction with eosinophilia and systemic symptoms, Stevens–Johnson syndrome, drug-induced liver injury, and simple rash, in comparison to 172 healthy individuals of their previous study [43]. While sjTREC levels still correlated with age, the association was weaker in individuals with compromised immune systems, with a mean absolute deviation (MAD) of approximately 11.59 when predicting the age of unhealthy individuals using the equation based on healthy individuals. Similarly, Farag et al. [49] collected blood samples from 233 Egyptian individuals divided into three groups, namely 90 with autoimmune diseases, 58 confirmed COVID-19 patients, and 85 healthy controls, and observed significant correlations between chronological and predicted age. In particular, MAD values were 9.40, 11.04, and 9.71 years for healthy individuals, autoimmune patients, and COVID-19 patients, respectively. Recognizing that aging is a complex process influenced by genetic, developmental, and physical factors, the accuracy of the assay might be further improved by an approach that uses multiple predictors derived from independent sources. For example, Cho et al. [50] firstly analyzed samples based only on sjTRECs analysis, obtaining a MAD value of 10.33 years, and then combined sjTRECs quantification with DNA methylation profiling on blood samples collected from 100 healthy Korean individuals, achieving highly accurate models, with correlations exceeding 96% and a MAD of approximately +3.31 years. Similarly, Elmadawy et al. [51] combined sjTREC levels with telomere length measurements on blood and buccal swab samples from 124 healthy Egyptian individuals, yielding stronger predictive accuracy (r = −0.876, *p* < 0.001) compared to either biomarker alone, leveraging the complementary decline of these markers with age. Considering only sjTRECs, the obtained SE was ±12.6 years. Overall, the data from this study provided enhanced evidence that human age estimation from blood using sjTRECs quantification is feasible at reasonably high accuracy and superior to telomere length estimation.

In conclusion, typical errors range from ±7 to ±13 years, with factors such as ethnicity, health conditions, storage duration, and detection methods influencing outcomes. Incorporating additional biomarkers, such as DNA methylation or telomere length, can further enhance predictions. Thus, sjTRECs quantification emerges as a promising tool for forensic and biomedical applications, with significant potential for refinement through ongoing research.

### 3.5. RNA Analysis

With advancements in molecular biology, genetics, and detection technologies, non-coding RNAs (ncRNAs), including microRNA (miRNA), circular RNA (circRNA), P-element-induced wimpy testis (PIWI)-interacting RNA (piRNA), along with mRNA, are increasingly being recognized for their significant potential in forensic medicine applications due to their potential to provide quite accurate and reliable information from biological samples [52]. The forensic utility of RNA is rooted in several key characteristics, including the stability of some RNA types, under specific environmental and experimental conditions, its high degree of tissue specificity, and its dynamic nature, as RNA expression patterns can change significantly in response to various factors such as age, environmental influences, and physiological states. These properties make RNA an invaluable tool offering insights into both the origin of biological samples (such as blood, saliva, or tissue samples, even in trace amounts or degraded conditions) and the temporal aspects of gene expression [53], helping in age estimation particularly when traditional morphological methods are unavailable or impractical. Numerous authors have investigated the various types of RNA, both independently and in combination with one another, as well as in conjunction with other age estimation techniques.

Messenger RNA (mRNA) is one of the most extensively studied RNA molecules. However, it is intrinsically less stable than DNA due to its susceptibility to degradation by ubiquitous ribonucleases (RNases) [54,55,56]. This RNA molecule serves as an intermediary between DNA and protein synthesis and it carries the genetic instructions encoded in DNA to the ribosomes, where this information is translated into specific proteins, playing a central role in gene expression [57]. Deng et al. [58] examined the relationship between the expression levels of ERCC1 and XPF mRNA (key enzymes in nucleotide excision repair, NER) and age in peripheral blood mononuclear cells (PBMCs) from 150 healthy Han Chinese individuals. After the RT-PCR, regression formulas were established to estimate age based on mRNA levels. The results showed that ERCC1 and XPF mRNA levels in PBMCs decline with age (ERCC1: r = −0.578; XPF: r = −0.844, *p* < 0.01), indicating a strong negative correlation. Zubakov et al. [59], instead, using genome-wide microarray analysis on peripheral venous blood collected from healthy males, identified 222 age-related mRNA markers, of which 9 were selected for validation. Among these, the *NRCAM* mRNA marker showed the strongest correlation with age. However, the general prediction model showed a MAD of approximately 9.20 and the markers were primarily downregulated with age, except for CFH, which was upregulated. In addition, they showed that combining mRNA with other biomarkers, such as DNA methylation markers, improved age prediction accuracy. A model including five DNA methylation markers and one mRNA marker (*NRCAM*) achieved a MAD of 4.60 years, suggesting that mRNA provides information that is independent of and complementary to that of DNA-based methods. Despite their lower predictive power compared to DNA methylation markers, mRNA markers hold promise when integrated into multimarker models for age estimation. One of the more recent studies, published by Dørum and colleagues [60], used RNA-Seq data to investigate potential RNA markers for age prediction in blood samples, including forensic-style dried blood stains. Two approaches were applied to select age-related markers: *DESeq2* differential expression analysis, which identified genes with expression changes linked to age, and machine learning with lasso regression, which optimized gene selection for predictive accuracy. Both methods produced lists of 270 candidate genes. To evaluate the predictive power of these genes, lasso regression was performed on both the *DESeq2*-derived and lasso-derived lists. In the US dataset (publicly available sequencing data), the lasso list incorporated 201 genes with an MAE of 4.35 years, compared to an MAE of 7.78 years for the 30 genes of the *DESeq2* list. In the RNAgE dataset (data containing five different sample batches), the lasso list included 77 genes with an MAE of 4.46 years, while the *DESeq2* list had 73 genes and an MAE of 12.1 years. Despite the promising results, the study faced challenges, including a reliance on public RNA-Seq datasets to supplement their limited sample size (117 dried blood samples) and the inherent variability of blood transcriptomes, which are influenced by environmental and tissue interactions. Additionally, saliva samples analyzed through RNA-Seq and miRNome profiling presented difficulties due to low RNA quality and a high proportion of microbiotic content, making them less reliable for forensic age estimation compared to blood.

Fang et al. [61,62] investigated the potential role of microRNAs (miRNAs), small non-coding RNAs with high stability (even in trace or stored bloodstain samples) and tissue specificity, for chronological age prediction. In a first study they analyzed the miRNA expression profiles of blood samples from Han Chinese individuals aged 20–69 years massive parallel sequencing (MPS). Six age-associated miRNAs (miR-98-3p, miR-324-3p, miR-32-3p, miR-330-5p, miR-374c-5p, and miR-342-3p) were identified through linear regression and Pearson correlation, with some showing positive and others negative correlations with age. An age prediction model was built using these miRNAs and the AdaBoost algorithm, achieving MAEs of 5.52 years for males and 7.46 years for females [61]. Subsequently, in a second study, instead, they aimed to investigate the potential of PIWI-interacting RNAs (piRNAs) and miRNAs for age estimation; piRNAs are small non-coding RNA molecules, 24–32 nucleotides in length, involved in the PIWI-piRNA pathway, a common route through which transposons are suppressed in most animals. By using MPS, five age-related piRNAs were identified in blood samples stored for eight years. Real-time PCR analysis confirmed that two piRNAs (piR-000753 and piR-020548) were effective for age estimation, alongside two miRNAs (miR-324-3p and miR-330-5p). A machine learning model using gradient boosting yielded the lowest MAE (approximately 3.17 years), demonstrating high accuracy in age estimation, with over 81% of the samples showing an error of less than five years, highlighting the potential of combining two different markers, especially for aged blood samples [62].

In 2022 Wang et al. published two studies [63,64] focusing on age estimation by analyzing circular RNAs (circRNAs), which regulate gene expression, via interaction with miRNAs and RNA-binding proteins as molecular sponges [65]. The first study [63] identified over 40,000 circRNAs in the blood of 13 healthy individuals aged 20–62 years using circRNA sequencing. By applying three statistical methods, namely false discovery rate, lasso regression, and support vector machine, 28 circRNAs were selected for validation in 30 additional individuals via RT-qPCR. From these, five age-related circRNAs were chosen to construct age prediction models using 100 blood samples from individuals aged 19–73 years. The regression tree and random forest regression (RFR) models achieved the highest accuracy in the testing subset, with MAEs of approximately 8.77 and 9.13 years, respectively. However, models performed better for younger individuals (under 40) and males, with an MAE of about 6.13 years in males compared to 10.92 years in females using the regression tree model.

In another study, Wang et al. [64] used a modeling set composed of blood samples from 200 volunteers aged 20–80 years (102 females and 98 males) to develop predictive models using miRNAs and circRNAs as biomarkers. They constructed models based on 15 ncRNAs (11 miRNAs and 4 circRNAs) using various machine learning techniques, including RFR and support vector regression. Among these, the RFR model demonstrated the best performance, achieving an MAE of 3.68 years in the training set and 6.84 years in the test set. Combining miRNAs and circRNAs resulted in better accuracy compared to using either biomarker alone. However, the study noted systematic prediction errors, with age being underestimated in older individuals and overestimated in younger adults. Additionally, both Wang’s studies presented the absence of children and adolescents in the sample set and significant sex effects, with models yielding less accurate predictions for females.

Finally, Salignon et al. [66] utilized 103 plasma samples from healthy individuals, aged 20 to 83 years, to assess age-related molecular changes in proteins and small RNAs. Proteomic analysis identified 21 age-associated proteins, with increased abundance of complement system components correlating with age. Small RNA sequencing revealed 315 age-altered small RNAs, primarily miRNAs, which showed a significant age-related decline and were predicted to target genes linked to growth, cancer, and senescence. Age-predictive models demonstrated that proteins were most accurate (R^2^ = 0.59), while miRNAs were the most predictive small RNA class (R^2^ = 0.54). Combining proteomic and miRNA data improved predictive accuracy (R^2^ = 0.70), underscoring their complementary roles in capturing age-related physiological changes.

### 3.6. Telomere Length Analysis

Specific DNA sections called telomeres are found at the ends of chromosomes. By employing a novel methodology, investigating these areas can yield data and enable us to calculate their chronological age since telomere length gradually decreases over time. This is precisely the reason why they can be used as biomarkers for the study of aging and chronological age. Telomere length has been analyzed and investigated in a number of studies in the literature using data from various biological sources, including blood, saliva, muscle, teeth, and dental pulp.

Elmadawy et al. [51] analyzed data from 248 buccal swabs, collected from 124 individuals (68 males and 56 females) aged from 0 to 78 years, to estimate the relationship between telomere length and assumed chronological age. They used the TeloTTAGGG telomere length assay and emphasized that length decreases with age, using a correlation index with a negative value. A correlation index with a value of 0.726 was the outcome of their subsequent combination of the findings from a multivariate model with the addition of extra molecular variables. The resulting mean prediction error was 12.5 years. These findings emphasize the necessity of integrating the outcomes of several biological parameters in order to improve forecast accuracy.

The research group of Srettabunjong et al. [67] processed 100 blood samples, collected from 50 males and 50 females, from fresh cadavers to explore the correlation between aging and telomere length. The results of this study showed that the decrease in telomere length is proportional to advancing age, resulting in a correlation index with a negative value. With a quadratic correlation score of 0.391, they were able to demonstrate the importance of appropriate sample conditions for increased accuracy and the necessity of starting samples of sufficient quality.

Differently, Magi et al. focused their attention on semitendinosus muscle biopsies. They included biopsies of 26 healthy males aged between 20 and 50 years. Their multiple regression analysis revealed that age is a significant independent variable associated with telomere length. Moreover, they observed that age is also independently correlated with oxidized environments and partially influenced by lifestyle habits [68].

Other studies have focused on the analysis of dental samples and the processing of dental pulp.

The study conducted by Márquez-Ruiz et al. [69] analyzed 91 permanent teeth from 77 individuals (20 men and 53 women) aged between 15 and 85 years, using quantitative PCR to measure telomere length. In this study, the data showed a negative correlation reflected in the shortening of the length with the progression of time and consequently with chronological age. However, the study obtained an MAE of approximately 9.85 years, highlighting the importance of integration between different methods in complex forensic cases.

Finally, Tejasvi and colleagues [70] used 30 dental pulp samples from individuals aged between 15 and 35 years, divided into three groups: 11–20 years, 21–30 years, and 31–40 years. Telomere length was measured by real-time quantitative PCR (qPCR), with the use of a standard curve to calculate absolute telomere length, resulting in a kilobase-level reduction ranging from 9.92 to 9.13 for the young and the elderly, respectively. However, the individual variability in the data demonstrated how important further standardization of methods is to ensure more robust results.

These studies highlight a convergence on the role of telomeres as useful biomarkers for age estimation. Telomere length remains a key parameter, with potential forensic and clinical applications. This is despite limitations related to interindividual variability and DNA quality. The next step towards improving the precision and reliability of these analyses could be integrating molecular techniques and more sophisticated statistical methods.

### 3.7. DNA Methylation

Many authors have identified CpG sites where methylation level is correlated with age and built age prediction models targeting small numbers of sites that can be incorporated into assays to estimate age with high accuracy, using technologies such as: Sanger sequencing, methylation-specific PCR (MSP), methylation-sensitive high-resolution melting (MS-HRM), MassARRAY, next-generation sequencing (NGS), single-base extension (SBE) using SNaPshot chemistry, pyrosequencing, and various other techniques. These techniques have mainly focused on whole blood, with some studies on other tissues such as saliva, semen, bone, and teeth [71].

Almost all the studies applied bisulfite conversion in order to discriminate between methylated and non-methylated cytosine [72]. It is required to generate DNA sequence variants; otherwise, methylated cytosine and non-methylated cytosine will be amplified with no alterations in the sequence during PCR amplification. When non-methylated cytosine is exposed to sodium bisulfite (NaHSO3), it deaminates and changes to uracil; instead, methylated cytosine remains intact, allowing their detection in downstream analysis [73,74].

#### 3.7.1. Sanger Sequencing

The Sanger sequencing technique has proven to be a useful tool for DNA methylation analysis for the prediction of chronological age. Through the study of this technique, it was possible to explore specific sites for chronological age prediction and the corresponding correlation in specific CpG sites on different biological matrices, as well as in multitissue predictive models, both in living and deceased individuals.

The research groups of Correia Dias et al. have, in fact, conducted several in-depth studies using the Sanger technique in order to predict age in forensic contexts [75,76,77]. Their first study [75] analyzed 70 blood samples from deceased individuals. The results obtained showed a strong correlation between methylation level and chronological age at the level of the *ELOVL2* gene (R = 0.785). Additional markers, such as *C1orf132*, *PDE4C*, *EDARADD*, and *FHL2*, were also studied to create a multivariate linear regression predictive model. This model obtained a MAD value of 6.08 years in the training set and 8.84 years in the test set. Their study also emphasized the applicability with the use of degraded starting samples, making the model suitable for postmortem samples.

The research group of Correia Dias et al. [76] also performed a study on samples obtained from living individuals. They analyzed 53 peripheral blood samples to study a set of four genes: *ELOVL2*, *FHL2*, *EDARADD*, and *PDE4C*. Their model yielded a MAD result of 5.35. The same model was validated through a pool of 18 additional samples that resulted in a MAD value of 4.98 years. Subsequently, the model was used for the analysis of an additional 51 cadaveric samples, resulting in a significantly increased MAD value of 9.72 years. The same research group [77] decided to extend the application of Sanger sequencing to multiple matrices with a follow-up study on a pool of 185 samples from blood, bone, and teeth. A combination of seven CpGs were analyzed for a multimatrix model, yielding results with significant levels of correlation between methylation level and chronological age, with a final MAD result of approximately 6.06 years.

Finally, the study by Khan et al. [78] conducted a pilot investigation using the Sanger sequencing technique on samples obtained from buccal swabs, analyzing the *NPTX2* gene in 26 individuals (13 male and 13 female) aged between 1 and 65 years. In contrast to previous studies, no significant correlations were found between methylation levels and the chronological age of the individuals. The study did, however, emphasize integrity at the technique level, affirming that the discrepancies may be attributable to the different biological matrix and not to methodological problems.

#### 3.7.2. Methylation-Specific PCR (MSP)

Analysis of DNA methylation level can be carried out through a technique called methylation-specific PCR (MSP), which has been used by numerous studies to estimate chronological age from different biological matrices.

The study by Cui et al. [79] analyzed eight CpG sites at the promoter level of the *hTERT* gene out of a total of 12 initial blood samples, then used 32 samples to validate the method. From the 12 blood samples, the MAD value of each CpG site ranged from about 4.29 to 5.95 years, showing small deviations. On the other hand, with regard to the set of 32 validation samples, the MAD level was slightly higher with a value of about 4.35 years, according to what was obtained from the test set.

Another study that considers the *hTERT* gene was conducted by Xin et al. [80] by combining traditional methylation-specific PCR with quantitative real-time PCR. The starting pool involved the use of 60 blood samples for model development and an additional validation set comprising 30 samples. The model based on methylation-specific PCR achieved a MAD value of approximately 6.60 years, while the result is slightly lower when the technique is paired with quantitative real-time models, reaching a MAD value of 5.19 years. These results have undoubtedly provided further insights into the methylation level of the *hTERT* gene promoter for age prediction in forensic contexts.

The real-time MSP technique was also applied by the research group of Kondo et al. [81] to study the methylation levels of the *ELOVL2* gene using samples obtained from the teeth of 29 individuals aged between 20 and 79 years. The analysis highlighted a significant level of correlation with a Pearson correlation coefficient of r = 0.843, which increases with the individual’s chronological age. Regarding the MAD value obtained, it was approximately 8.94 years, due to the greater variability in older subjects.

The study by Ogata et al. [82] also used methylation-specific PCR technique with real-time analysis by analyzing CpG sites at the level of two genes: *ELOVL2* and *EDARADD*. Ninety-nine teeth from Japanese donors were analyzed. The samples were divided into a training group with a total of 59 samples and the remaining 40 samples constituted the test set. The *EDARADD* gene showed a correlation with a negative value while the *ELOVL2* gene showed an opposite trend with a positive correlation value. The multiple regression model combining both genes improved the accuracy of the estimate, with an MAE of 6.69 years in training samples and 8.28 years in test samples. However, accuracy was slightly lower in older individuals than in young and middle-aged individuals.

The study conducted by Soedarsono et al. [83] represented another step forward in the application of MSP, integrating it into a broader context to develop prediction models based on epigenetic age. They analyzed three genes in a sample pool from 43 teenagers of Indonesian origin between the ages of 11 and 20 years. The *ELOVL2* gene showed a strong correlation between methylation level and chronological age (r = 0.964), with a positive value emphasizing the increase in methylation levels with advancing age. With regard to the other two genes (*PRKG2* and *EDARADD*), the Pearson correlation index values obtained are r = −0.955 and r = −0.942, respectively. In contrast to *ELOVL2*, these two values are negative, meaning that the methylation level decreases with increasing chronological age of the individual.

#### 3.7.3. Methylation-Sensitive High-Resolution Melting (MS-HRM)

Methylation-sensitive high-resolution melting (MS-HRM) is a method that measures methylation profiles, where the PCR amplification of bisulfite-treated DNA is followed by melting analysis. Therefore, the methylation status of each cytosine is directly converted into the sequence, where it alters the thermodynamic stability of double-stranded DNA, distinguishing methylation profiles through changes in the melting curve. In forensics, it can be used for age prediction at the level of related genes. This technique can be applied starting from different biological matrices such as saliva, blood, and buccal cells but also extending to less conventional samples such as cigarette butts, highlighting the method’s flexibility.

Several studies in the literature demonstrated the flexibility of the technique itself. Oka et al. [84] studied salivary samples from 79 voluntary donors of Japanese origin and 34 donors of Indonesian origin, comparing these different populations. The work analyzed the methylation levels of the genes *ELOVL2*, *EDARADD*, and *FHL2*. The accuracy of the analysis is ensured by the premelting and postmelting temperatures specific to each gene. The methylation levels of the *EDARADD* gene in particular showed significant divergences in the two populations. The MAD value obtained considering all samples was 11.1 years, while the MAD value obtained when the model was settled, distinguishing the two populations, was 10.8 years. This study was able to show that, for groups with a higher age, the ethnicity factor flattens out more and more, thus resulting in a lesser impact on methylation analysis. However, limited information is available on whether these methods are accurate across different populations.

Finally, the research group of Hamano et al. [85,86] analyzed both salivary and blood samples by the MS-HRM technique, conducting two different studies. The first study considered 74 blood samples: 22 were from living individuals and the remaining 52 from deceased bodies (aged between 0 and 95 years), analyzing the two genes *ELOVL2* and *FHL2* to try to obtain a predictive model combining both living and deceased individuals. The model validation, evaluating both genes simultaneously, obtained a MAD value of 7.44 years. They also performed an analysis by gender that did not lead to significant differences, underlining how the method may be universally applicable [85]. In the second study, they analyzed 263 saliva samples from healthy individuals and samples taken from cigarette butts donated by 16 volunteers; the donors ranged in age from 1 to 73 years and the methylation levels of the two genes *ELOVL2* and *EDARADD* showed linear correlations with age. The chronological age prediction model obtained a MAD value of 5.96 years in the training set and a range of approximately 6.25 years in the validation set. The highest accuracy is found in the age range of younger individuals, consequently the analysis is less reliable when dealing with older individuals. The application of the method to a non-conventional biological matrix such as cigarette butts obtained, after data analysis, a MAD value of 5.64 years [86].

#### 3.7.4. MassARRAY and Microarray

The MassARRAY technique is based on the study of DNA methylation using mass spectrometry, usually matrix-assisted laser desorption/ionization time-of-flight (MALDI-TOF), to quantitatively analyze the methylation of CpG sites by specifically cutting the bases at particular sequences, allowing the prediction of chronological age by analyzing biological samples such as teeth, blood, or saliva. This technique thus makes it possible to identify changes that occur on the DNA at precise positions such as methylation of the strand itself in particular regions that are thus age-related. Although MassARRAY can be used as a standalone method to examine methylation at specific regions, as performed by Giuliani et al. [87], Yi et al. [88], and Freire-Aradas et al. [89], it could be flanked with microarray techniques. For instance, Freire-Aradas et al. [90,91] and Zubakov et al. [59] used both these techniques. In this context, microarray has been used to identify the most relevant CpG sites associated with age, while MassARRAY has been applied in the subsequent validation analysis. However, microarray can also be used independently, as performed by Aanes et al. [92] and Bocklandt et al. [93], allowing analysis of DNA methylation using specific probes for genomic sequences. It measures the level of methylation of CpGs in different areas of the genome, providing information on epigenetic changes in various biological samples.

The research group of Giuliani et al. [87] used a MassARRAY technique to analyze DNA methylation levels in teeth. In particular, they analyzed DNA samples extracted from 22 human teeth from individuals aged between 17 and 77 years. The results obtained a MAD value of 1.20 for the combined pattern of DNA extracted from the pulp and cementum of the starting teeth by analyzing eight CpG sites in the *ELOVL2* and *FHL2* genes. The authors underlined the great potential of the technique in the forensic field with samples of dental origin considering the great precision obtained.

Similarly, Yi et al. [88] used a MassARRAY technique to study age–methylation correlation analysis and age prediction with blood samples obtained from 65 volunteers aged between 11 and 72 years. The eight isolated fragments associated with human genes were: *TBOX3*, *GPR137*, *ZIC4*, *ZDHHC22*, *MEIS1*, *UBE2E1*, *PTDSS2*, and *UBQLN1*. The CpGs showed significant variations in methylation levels between the different age groups. The multivariate regression model obtained very high Pearson correlation indices, underlining a strong accuracy with regard to the ultimate aim of age prediction, with values of r around of 0.91. Additional checks were also performed on separate male and female models with correlation index accuracy of R^2^ = 0.889 and R^2^ = 0.971, respectively.

The research group of Freire-Aradas et al. conducted three different studies using MassARRAY alone or preceded by microarray application [89,90,91]. The first study [90] used the EpiTYPER^®^ system to analyze a total of 725 blood samples from individuals aged between 18 and 104 years. They also studied samples from 52 monozygotic twin pairs aged between 42 and 69 years. They selected 18 CpG sites correlated with age and, using microarray analysis, the model developed obtained a correlation level of R^2^ = 0.9395 in the training set. The median absolute deviation value was 3.07 years, while for the model built with samples from monozygotic twin pairs, the obtained MAD value was 4.23 years. Similarly, in a second study [91] they applied MassARRAY preceded by the microarray technique to identify CpG sites and analyze a total of 209 samples from European donors aged between 2 and 18 years with approximately 10 samples per age. Some genes, such as *KCNAB3*, *PRKG2*, *EDARADD*, *ELOVL2*, *FHL2*, and *MIR29B2CHG*, were analyzed to assess the correlation between DNA methylation and age in children and adolescents. Thanks to the predictive models obtained, the MAE value was 0.94 years in the training set and 1.25 years in the validation set, conducted on a total of 29 independent samples. The *KCNAB3* gene proved to be a highly informative biomarker for these particular age groups. The third study [89], instead, was focused only on MassARRAY. A pool of 895 blood samples for the training set from Spanish donors aged between 2 and 104 years, followed by a further pool of 152 samples to validate the previously created model, were considered. The ages of the test set are between 3 and 69 years. A total of 1047 samples were therefore analyzed for the seven reported genes: *ELOVL2*, *ASPA*, *PDE4C*, *FHL2*, *CCDC102B*, *MIR29B2CHG*, and *chr16:85395429*. The study conducted analyzed three different statistical models in parallel: quantile regression (QR), quantile regression neural network (QRNN), and quantile regression support vector machine (QRSVM). They obtained different mean error values, the best results being obtained with the QRNN and QRSVM of 3.36 and 3.41 years for the training test and 3.32 and 3.45 years for the validation test, respectively.

The study conducted by Zubakov et al. [59] analyzed a total of 216 blood samples from individuals aged between 4 and 82 years. The results of their study obtained a highly significant correlation of R^2^ = 0.931 by analyzing 43 methylation markers validated with EpiTYPER technology. The MAD for this studied model was found to be 4.23 years.

Aanes and colleagues [92] analyzed a pool of 973 samples aged 12 to 25 years for a narrow age span and a group of 2316 samples for a broad age span (10 to 60 years) using the microarray technique. The training set for the narrow dataset resulted in better test performance with a MAD value of 0.73, but predictions were mostly around 1 year and just a few samples showed an error of more than 3 years. The Pediatric Age from Young Adults (PAYA) model utilized showed the highest precision and accuracy compared to other models.

Finally, similarly to the previous study, Bocklandt and co-workers [93] used the microarray technique alone. They analyzed salivary samples of 34 male identical twin pairs aged between 21 and 55 years. Then, they analyzed, to validate the model, another group of 60 samples (31 males and 29 females, aged between 18 and 70) and the promoters of three genes, *EDARADD*, *TOM1L1*, and *NPTX2*, using Illumina HumanMethylation27 microarrays, combined with statistical analysis leave-one-out methods to generate a multivariate regression model. They obtained different average accuracies to predict an individual’s age of 5.3 years for the male-only model, 6.2 years for the female-only model, and a value of 5.2 years for the combined sample.

#### 3.7.5. Next-Generation Sequencing (NGS)

Next-generation sequencing (NGS) techniques, including Illumina BeadChip, massively parallel sequencing (MPS), and whole genome sequencing (WGS), have been applied to study methylation levels for chronological age prediction due to their ability to process a great number of genes and data compared to other techniques.

The working group of Zhang et al. [94] analyzed 1545 blood samples from healthy individuals and rheumatoid arthritis patients. They obtained a MAD value of 3.90 years in healthy subjects and 3.11 years for AR-affected individuals, analyzing 24 CpG sites. They observed specific variations in disease conditions, but not impacting on the overall regression model, still ensuring reliable results.

The longitudinal study by Refn et al. [95] similarly analyzed blood samples with the difference that they were collected at two different times 15 years apart. A total of 128 blood samples from 64 voluntary donors were used. This study evaluated the stability of the markers over time, showing that ELOVL2 is particularly suitable and efficient from this point of view.

Vidaki et al. [9] also analyzed 1156 blood samples from living individuals aged 2–90 years. They also analyzed: 265 salivary samples, 46 blood samples for model validation, 53 pairs of single-zygote twins, and 1011 samples from diseased individuals. In this study they used the artificial natural network (ANN) model, obtaining an MAE of 3.3 years for the training test and 4.4 years for the blind control test on a set of 16 analyzed CpG sites.

The study by Amiri Roudbar et al. [96] analyzed over 485,000 CpG sites with data from four public datasets considering 4409 individuals. The results have led to significant and robust correlation indices and have shown differences in gender, highlighting that the epigenetic aging rate in men presents methylomic changes faster than that of women.

When considering saliva samples, the study by Hong et al. [97] highlighted highly predictive markers for age, identifying 62 CpG sites that can be used through a linear univariate regression model. They also used additional models such as stepwise regressions that led to the identification of a MAD value of about 3.83 years on a lower CpG site pool.

The study by Lau and Fung [98] processed data from 991 blood samples using multiple linear regression models and machine learning models, analyzing a pool of 16 selected markers and obtaining a MAD of about 3.74 years.

Alsaleh and Haddrill [99] processed data from the study of 754 samples aged 0–88 years. After a series of experiments, they tried to study a simplified model that includes six marker genes and which obtained a MAD value of 4.5 years for the training test and 4.6 years for the validation set. Instead, using methylation profiles to develop a simplified model on six CpG markers, they achieved a MAD of 4.6 years in validation tests.

Lee et al. [100,101] have conducted two studies to refine the technique with a pool of different samples. In the first study, they analyzed 157 semen samples from individuals of Korean origin aged between 18 and 70 years. They also added additional samples of different biological origin, such as three blood samples from female individuals and three vaginal fluid samples, in order to be able to carry out comparisons between genders. The study used MPS and several regression models such as stepwise, linear multivariate regression, elastic net regression, lasso regression. As for the results of model validation in the various regression systems, they obtained MAE values ranging from 4 to 7 years. In the second study, different bone samples were analyzed [100]. Data from samples of 66 identified bone remains with an age at death ranging from 31 to 96 years were processed. The samples were halved in the following groups. Preliminary tests were carried out on four samples. Of the remaining 62, 30 were analyzed with the Illumina BeadChip technique while the last 30 were used for model validation. The validation samples showed a moderate age correlation in only four genes (*TMEM51*, *TRIM59*, *ELOVL2*, *EPHA6*), underlining how complicated and challenging it is to choose appropriate markers for age prediction [101].

Similarly, the study by Jung et al. [102] focused on the analysis of samples of cartilage origin taken from 85 individuals, mostly men, aged between 26 and 89 years. The work analyzed MPS data from nine genes considered: *TMEM51*, *MIR29B2CHG*, *EDARADD*, *FHL2*, *TRIM59*, *ELOVL2*, *KLF14*, *ASPA*, and *PDE4C*. The multivariate stepwise linear regression model obtained an MAE value of 4.17 years while for the validation set the MAE value increased slightly to a value of 4.97 years. The study also showed a high sensitivity even from small amounts of DNA (optimum value around 10 nanograms, with reliable results up to 5.5 nanograms).

The research group of Hong et al. has conducted two different studies using the MPS technique [103,104]. In the first study, they analyzed as a test set saliva samples from 96 donors aged between 18 and 65 years, half male and half female. The results revealed strong correlations between DNA methylation at specific CpG sites and age and, for the *cg00481951*, *cg07547549*, and *cg14361627* sites, the values of Pearson’s correlation coefficients were found to be between 0.756 and 0.814. They obtained a very high MAD value of around 23.42 years, significantly higher than reported in the literature by other studies [103]. The second study analyzed samples from blood samples belonging to 250 donors of Korean origin, perfectly divided between males and females, aged between 20 and 74 years considering the methylation level in *ELOVL2*, *FHL2*, *KLF14*, *MIR29B2CHG*, and *TRIM59*. The obtained results showed strong correlation indices for all CpG sites analyzed [104].

Guan and coll. [105] analyzed postmortem blood samples of 90 Japanese individuals aged from 2 weeks to 91 years. Samples were divided into a training dataset (60 samples) and an independent validation dataset (30 samples). From a pool of 11 genes the four CpG sites *ASPA*, *ELOVL2*, *ITGA2B*, and *PDE4C* showed the strongest correlation with chronological age. *ELOVL2* was identified as the most informative marker. The linear regression model gave a MAD of 5.23 years (training dataset) and 6.49 years (validation set).

Becker et al. [106] sampled a piece of the left parietal bone obtained from skull bone of 190 cadavers (aged 0–96 years, 55 females and 135 males) by multiplex PCR amplification targeting genomic regions of six age-associated genes: *ELOVL2*, *KLF14*, *PDE4C*, *RPA2*, *TRIM59*, and *ZYG11A*. The DNA methylation markers exhibited significant age-related changes with high Spearman’s correlation coefficients ranging from 0.79 to 0.93 across the markers. Regarding the cross-validation performance, DNA methylation markers yielded the best results with an MAE of 4.95 years.

Woźniak et al. [107] analyzed 160 blood samples (1–75 years) sourced from healthy volunteers, 160 samples of buccal cells (2–80 years) from forensic genetic archives, and 161 bone samples (19–93 years) collected during routine autopsies. The study used a multiplex PCR assay for eight markers (*ELOVL2*, *PDE4C*, *MIR29B2CHG*, *KLF14*, *FHL2*, *TRIM59*, *EDARADD*, and *ASPA*) showing high accuracy with minimum DNA input (20 ng) for reliable methylation quantification. Strong age correlation was observed in CpG sites across all tissues, with variations noted in older age groups.

Regarding accuracy, the reported MAEs were 3.2 years for blood, 3.7 years for buccal cells, and 3.4 years for bone tissues. Aliferi et al. [108] analyzed 110 samples of whole blood (donor ages ranged from 11 to 93 years), used for initial model development, validation, and blind testing, 34 samples of saliva analyzed for model applicability, and 11 semen samples specifically focusing on the sperm fraction. The genes investigated were *VGF*, *TRIP10*, *KLF14*, *CSNK1D*, *FZD9*, *C21orf63*, *SSRP1*, *NHLRC1*, *ERG*, *FXN*, *P2RXL1*, and *SCGN*. Sensitivity testing for all samples demonstrated reliable predictions down to 10 ng of input DNA, but accuracy decreased significantly at 1 ng of input DNA. In particular, for saliva, successful age prediction was achieved with a mean error of 7.3 years despite the model being trained on blood, indicating potential for cross-tissue applicability. Notably, no methylation was detected in the sperm fraction. The best performance was obtained with the statistical model support vector machine with an MAE of 4.1 years in the blind test set.

Naue and co-workers [109] analyzed blood samples of 324 individuals (208 for the training set, 104 for the test set), with the age range of 18–69 years and an equal gender distribution. The study developed a DNA-methylation-based model for age prediction using 13 specific CpG markers. An RFR model was used for age prediction and achieved a MAD of 3.16 years and, among the 13 genes analyzed, the four markers ELOVL2, F5, TRIM59, and KLF14 contributed most to the performance of the model.

In another study, Naue et al. [110] included 29 individuals selected based on age and gender, with an equal distribution of males and females of different ages. A total of 144 samples were collected from various tissues, including blood, skeletal muscle, brain, bone, and buccal swab, and analyzed using the MPS technique on the following genes: *RPA2*, *F5*, *TRIM59*, *KLF14*, *HOXC4*, *NKIRAS2*, *ZYG11A*, *MEIS1*, *ELOVL2*, *GRM2*, *LDB2*, *SAMD2*, and *DDO*. In this pilot study, the authors concluded that a larger sample set is required to create and validate tissue-specific models for DNA methylation age prediction. They also suggested that the most informative CpG sites for each tissue should be identified. Tissue-specific age prediction models will be valuable, and when the tissue origin of a sample is known, not all markers need to be measured.

Ochana et al. [111] analyzed DNA from blood samples obtained from 300 healthy donors across three independent cohorts. They studied a pool of CpG sites and demonstrated that *C1orf132*, *CCDC102B*, *ELOVL2*, and *FHL2* present the best MAEs of approximately 2.36, 2.98, 1.65, and 3.21 years, respectively. In particular, for individuals under 35 or 50 years of age the model reached a higher accuracy. Their results showed that, in order to achieve an optimally performing model it is important to involve both a locus that can change methylation with time, such as *ELOVL2*, and a locus that can reflect the age-related characteristic alterations in cell composition, such as *C1orf132*.

Recently Refn and collaborators [112] published a study on an 11-CpG panel for age estimation in 148 blood samples obtained from donors aged between 18 and 68 years, equally divided into males and females. The panel was composed with two multiplexes constructed with the following genes: *FHL2*, *ARHGAP22*, *TRIM59*, *CCDC102B*, *CNTNAP2*, *LDB2*, *ELOVL2*, *RASSF5*, *MIR29B2CHG*, and *EDARADD*. Results showed that the DNA methylation at the 11 CpG loci was significantly correlated with age in the sample set, confirming the potential of the 11 CpGs in age prediction. The applied support vector machine with polynomial function model (SVMp) was retrained using data from 108 of the 148 individuals, while data from the other 40 individuals were used for validation. The MAE was 2.58 for the training dataset, while the MAE was 3.35 for the test dataset.

An important interlaboratory project was subsequently carried out within the VISAGE project by various studies such as Heidegger et al. [113], Pisarek et al. [114], Freire-Aradas et al. [115], Piniewska-Róg et al. [116], and Pośpiech et al. [117]. The VISAGE project has developed advanced tools for age prediction based on DNA methylation, using different analytical techniques and models applied to different biological matrices [118].

If we analyze these results in detail, we find the study of Heidegger et al. [113] in which they processed data obtained from two samples of seminal DNA and the technique of MPS. The 13 candidate CpG sites were studied in different models including ET-13 and ET-5, which obtained MADs ranging between 4.1 and 5.5 years.

Another study on semen was that of Pisarek et al. [114]. They validated different genes starting from 381 sperm samples. The final model based on six CpGs (*SH2B2*, *FOLH1B*, *EXOC3*, *IFITM2*, and *GALR2* in cytosine 1 and cytosine 3) obtained an MAE value of 4.3 years in the training set and 5.1 years in the test set. The specificity of methylation in sperm compared to somatic cells demonstrated the potential of this array for forensic applications.

The research group of Freire-Aradas et al. [115] developed a model from 181 samples of costal cartilage, studying a pool of 44 CpGs in the *KLF14*, *TRIM59*, and *FHL2* genes. The model after data processing obtained an MAE value of 4.26 years.

Piniewska-Róg et al. [116] conducted a study on 212 blood samples, differentiated for chronic alcohol abusers (106 samples) and controls (106 samples), using the VISAGE Age Tool. Methylation in the *MIR29B2CHG* and *FHL2* genes highlighted significant alterations in abusers, resulting in an MAE of 3.1 years compared to an MAE of 3.3 years in non-abusers.

Finally, the study by Pośpiech et al. [117] analyzed five blood samples from individuals of Polish origin aged between 7 and 78 years. The main results showed high sensitivity and adequate reproducibility of the technique with a significant correlation between DNA methylation levels and chronological age. In the comparison of three sequencing technologies, out of a total of 161 CpGs in association with four different epigenetic models, they showed that the best accuracy with an MAE value of 2.7 years was associated with the VISAGE model considering Ion AmpliSeq.

Another study performed on blood samples was that of Vidaki et al. [119]. They studied 1057 blood samples from males aged 15–87 years, sourced from six publicly available Gene Expression Omnibus (GEO) datasets: 758 samples for model training and 172 samples for validation, while the sixth dataset, comprising 127 samples, was used for independent testing, with the purpose of validating the applicability of Y-CpGs for male-specific age prediction. Using supervised machine learning, SVM, and RFR, the study developed a Y-CpG-based age prediction model. The SVM radial model, which accounted for non-linear age–methylation patterns, achieved the best performance with a MAD of 7.54 years in the validation dataset and 7.61 years in the testing dataset.

Llobet and collaborators [120] analyzed 811 blood samples, 377 males and 434 females, divided into three groups by age: 257 “younger” samples with an age below 40 years, 211 “middle-aged” samples aged 40–55 years, and 343 “older” samples aged above 55 years, constructing a linear regression and a stepwise model using the NGS technique. The combined model showed significant correlations between chronological and predicted ages. In the training set they obtained a value of R = 0.99 for both women and men and, for the test sets, a value between 0.95 and 0.99. They also displayed a standard deviation error between 3.3 and 6.5 years.

Finally, in the study conducted by Mawlood et al. [121], mitochondrial DNA (mtDNA) samples obtained from 82 female donors aged between 18 and 91 years were studied. The study focused on 10 regions of interest in mtDNA, which include both functional gene sequences and promoter regions. The analysis was performed using the NGS Illumina MiSeq technique. Hypomethylation levels were detected at two different CpG sites in the MT-RNR1 gene and there was a significant correlation with age. The linear regression model conducted obtained a MAD value of 9.3 years. The accuracy of this predictive model was highest in the 40–59 age group. The study showed that there are intrinsic and external factors that are able to influence mtDNA methylation as a biomarker for aging.

#### 3.7.6. Single-Base Extension (SBE)—SNaPshot

The SNapShot technique is based on single-nucleotide extension, called single-base extension (SBE). This technique uses the final aid of methods for reading, such as capillary electrophoresis, by means of different models of sequencers available on the market and currently used in forensic genetics laboratories, and for this it is used for the study of methylation level with the ultimate purpose of predicting chronological age in forensically relevant biological samples.

Regarding publications that consider blood samples, the study by Onofri et al. [122] analyzed 84 blood samples from Italian volunteers aged between 18 and 65, the level of methylation of five genes in a multivariate linear regression model, specifically *ELOVL2*, *FHL2*, *KLF14*, *C1orf132*, and *TRIM59*, and an additional four-gene model, excluding the TRIM59 gene. For both models the value of the absolute median deviation is about 3 years.

The same cluster of genes was considered by Grignani et al. [123] who analyzed 72 blood samples from living individuals aged 18–85 years and 29 samples from charred human remains. The data produced a MAD value of about 6.92 years for samples taken postmortem from carbonized individuals.

Similarly, the study conducted by Dias et al. [124] analyzed 59 blood samples from living Portuguese individuals and 62 samples from cadavers, evaluating the methylation value of the same five genes, reporting that the model of only three genes (*ELOVL2*, *FHL2*, and *C1orf132*) obtained the best results, identifying a value of MAD of about 4.25 years for living individuals and about 5.36 years for samples from the deceased.

A further study of blood samples was conducted by the research group of Han et al. [125] on a pool of 529 samples, of Chinese origin, aged between 2 and 82 years, analyzing eight different CpG sites and comparing several models such as multivariate linear regression and support regression vector, obtaining an MAE value of 3.52 years and 2.88 years, respectively. The results demonstrated a greater accuracy with the use of the second technique in the training test set.

Ye et al. [126] tested blood, fresh bloodstain, and aged bloodstain samples derived from 115 training samples of healthy individuals aged 11–71 years and 30 testing samples of healthy individuals aged 22–51 years for *ELOVL2*, *C1orf132*, and *DNAH9*, obtaining the following MAD values: blood: 4.73 years, fresh bloodstains: 4.49 years and aged bloodstains: 5.43 years. The prediction model was consistent across blood and bloodstains, with a slightly higher deviation for aged bloodstains.

Filoglu et al. [127] analyzed whole blood samples obtained from 100 healthy Turkish volunteers in the age range of 20–83 years, consisting of 40 females and 60 males: 40 individuals were selected for the test group, while the remaining 60 constituted the training group. A panel of eight genes were tested: *ELOVL2*, *FHL2*, *ASPA*, *C1orf132*, *CCDC102B*, *TOM1L1*, *RASSF5*, and *cg07082267*. Methylation profiles were consistent down to 1 ng of DNA, but errors increased at 0.5 ng due to dropout events at some loci. The MAE was 3.75 years, while the six-CpG model achieved an MAE of 4.07 years.

Jiang et al. [128] considered 186 male blood samples (ages 15–87 years) from a Han Sichuan population. A total of 13 Y-CpGs markers were investigated, obtaining with the multivariate linear regression a MAD of 12.36 years for the training test. Removing three non-significant loci, the MAD was reduced to a value of 10.34 years. The RF model resulted in a MAD of 4.65 years. In particular, the RF model was validated through sensitivity and mixed-sample tests showing successful performance with DNA inputs as low as 0.5 ng.

Pan and co-workers [129] analyzed 310 blood samples collected from Han Chinese volunteers aged 2–86 years, randomly divided into a training set of 230 samples (male 142, female 88) and a validation set of 80 samples (male 47, female 33), with *CCDC102B*, *ITGA2B*, *EDARADD*, *ZNF423*, *KLF14*, and *ASPA*. *CCDC102B* had the highest correlation with age, while *ZNF423* had the lowest. Significant differences in DNA methylation and age correlation were observed between the Han Chinese population and European populations (e.g., French, Belgian, Spanish, Polish). Moreover, they obtain a MAD of 4.22 years using a stepwise regression model and of 4.01 using a support vector model.

When considering saliva samples, Marcante et al. [130] developed an age-specific predictive model for salivary samples; when inserting data into a previously validated predictive model, calibrated on blood samples, the value of MAD was found to be about 10.28 years. In the new model, which studies the level of methylation of the five genes *ELOVL2*, *FHL2*, *KLF14*, *C1orf132*, and *TRIM59*, the value of MAD, by inserting the data obtained from the analysis of 60 salivary samples, was considerably lowered to about 3.49 years.

The study conducted by Carlsen et al. [131] also explored samples of oral origin, and 230 samples from volunteers aged between 1 and 88 years were analyzed, evaluating the differences between groups of adults and children by studying the following eight genes: *PDE4C*, *EDARADD*, *SST*, *KLF14*, *ELOVL2*, *FHL2*, *C1orf132*, and *TRIM59*. The study was conducted using the AB3130 sequencer to perform capillary electrophoresis. The data collected led to a MAD of 4.68 years for the training set and an almost equal result of 4.70 years for the validation set of the seven-gene model (*PDE4C*, *EDARADD*, *KLF14*, *ELOVL2*, *FHL2*, *C1orf132*, *TRIM59*). Overall, the highest accuracy was found for the younger age group.

Takahashi et al. [132] analyzed buccal swabs, studying 51 healthy Japanese individuals aged from 21 to 68, for *EDARADD*, *SST*, and *KLF14* genes, targeting specific CpG sites, using thermostable locked nucleic acid (LNA) primers to improve stability and amplification efficiency. For *EDARADD* and *SST* genes, LNA primers enhanced signal intensity, especially with small DNA quantities, achieving up to 3.3-fold improvement for *EDARADD* and 1.4-fold for *SST*. Age prediction was more accurate with LNA primers, yielding a MAD of 3.88 years, and when using 10 ng of DNA. Methylation levels of *EDARADD* were inversely correlated with age, while *SST* and *KLF14* showed positive correlations.

Another study performed on buccal swabs was that of Ambroa-Conde et al. [133] which analyzed a total of 368 saliva and buccal cell samples obtained from 184 volunteers of Spanish origin aged between 21 and 86 years. The study showed that, although saliva and buccal cells show similar patterns, the combination of tissue-specific markers significantly improves prediction.

The working group of Jung et al. [134] analyzed 448 samples from blood samples and saliva samples, studying in a Korean population the differences between different biological matrices in the same pool of five genes using blood from previous studies (*ELOVL2*, *FHL2*, *KLF14*, *C1orf132*, and *TRIM59*). The results of the combined model of the different biological matrices analyzed have obtained a MAD value of about 3.84 years.

Similarly, So and Lee [135] analyzed the same five genes in 132 samples, comprising 66 blood samples and 66 saliva samples, collected from individuals aged 18–69 years. The DNA samples were obtained from both sexes, with blood samples including 32 females and 34 males and saliva samples evenly split at 33 males and 33 females. Significant differences in methylation levels between the analyzers were statistically validated using the Friedman and Wilcoxon signed-rank tests. Despite the differences in methylation values, all CpG sites displayed a strong correlation with age, with correlation coefficients across all analyzers. In particular, *ELOVL2* showed the strongest correlation with age in blood samples.

Among the selected literature, only one study considered hair as a biological source for age prediction. Hao and co-workers [136] analyzed a total of 166 hair samples, 130 scalp hairs from donors aged 1 to 86 years, 6 pairs of black and white scalp hairs from six volunteers, and 24 hairs from four body areas (scalp, calf, pubic area, and armpit) from six volunteers. Donors were from a Han population in Shanxi Province, northern China. Ten CpG sites from eight genes (*LAG3*, *SCGN*, *ELOVL2*, *KLF14*, *C1orf132*, *SLC12A5*, *GRIA2*, *PDE4C*) were identified as age predictors. No significant differences in methylation were observed between male and female donors or among hair types (scalp, calf, pubic, armpit) and colors (black, white). Methylation patterns were consistent across hair samples. Regarding sensitivity, DNA from a single scalp hair follicle was sufficient for age prediction with an optimal input for reliable results of 4 ng of converted DNA. The resulting MAD was 3.68 years, while the validation on 40 testing samples confirmed strong performance with a MAD of 4.15 years.

Regarding semen fluid as source for age estimation, Lee HY and co-workers [137] conducted a preliminary study on semen samples that were collected from 94 healthy male volunteers aged 20–73 years, considering three CpG sites: *cg06304190* in the *TTC7B* gene, *cg06979108* in the *NOX4* gene, and *cg12837463*. These CpG sites displayed strong age associations and showed significant differences in methylation levels with age progression. In the training set, for 31 semen samples, the model achieved a MAD of 4.2 years, whereas in the test set of 37 samples the MAD value was 5.4 years. Larger deviations were noted in individuals aged over 60 years, potentially reflecting differences in biological versus chronological age. Compared to other tissue-specific models, such as blood, the semen-based model performed comparably, despite using fewer CpG markers.

The study of Lee JW et al. [138] is the first report of an age-predictive model for semen. They analyzed semen samples collected from 12 male voluntary participants, aged 24–57 years, and 19 DNA samples from forensic evidence. Reliable results were obtained with >5 ng of bisulfite-converted DNA, even at lower amounts (as little as 5 ng), though accuracy decreased at 1.25 ng or less due to allelic dropout. Performance was evaluated across four age groups (20s, 30s, 40s, 50s) and prediction accuracy decreased with increasing age from the 20s group (MAD = 2.9 years) to the 50s group (MAD = 7.2 years). Collectively, the accuracy of the age-predictive model for semen was 4.8 years, whereas for forensic evidence the MAD was 5.2 years.

Er et al. [139] present a study on a novel age estimation model based on semen-specific CpG methylation patterns, applied to ejaculate samples from 115 male patients diagnosed with infertility, age ranging from 20 to 71 years. The twelve targeted CpG sites included *PARP14*, *C5orf25*, *cg23488376*, *MXRA5*, *PFKFB3*, *DLL1*, *NOX4*, *cg12837463*, *TTC7B*, *KCNA7*, *NKX2-1*, and *SYNE4*. Most CpG sites exhibited a negative correlation between methylation levels and age, except for *NOX4*, which showed a positive correlation. This model incorporated all 12 CpG sites and achieved an MAE of 3.81 years.

Xiao et al. [140] reported DNA methylation data from 90 sperm and paired whole blood samples for donors aged 22–51 years, with validation performed using a larger cohort of 253 sperm samples (22–67 years) and an additional 40 blood samples, obtained from the analysis of 31 novel sperm-specific AR-CpG markers with strong age correlations, also distinguishing sperm/semen from blood samples with significant methylation differences. Age estimation accuracy for sperm was improved using the identified markers, particularly when combined into multimarker models, such as the 14-CpG model with an MAE of about 2.9 years. Age estimation accuracy decreased with increasing chronological age, as reported in previously reported articles.

The study of Lee JE and collaborators [10] evaluated age prediction models for three body fluids. A total of 24 test samples (saliva, blood, and semen) were prepared for a collaborative study of twelve laboratories. Body fluid typing from each laboratory provided sufficient information to determine appropriate age prediction methods. The SNaPshot multiplexes for age prediction used were: *ELOVL2*, *FHL2*, *KLF14*, *C1orf132/MIR29B2C*, and *TRIM59* genes in blood and saliva and *tcg06304190* in the *TTC7B* gene and *cg12837463* and *cg06979108* in the *NOX4* gene in semen. They reported the following values of MAE: for semen samples, MAE of 2.7 years; blood samples presented a higher error, with an MAE of 5.0 years, and age prediction for saliva samples demonstrated an intermediate MAE of 3.8 years. The collaborative exercise revealed significant variability in DNA methylation measurements among laboratories.

A similar study was conducted by So and collaborators [141] who evaluated the DNA methylation level in 362 samples, composed of 150 blood samples, 150 saliva samples, and 62 semen samples and an additional 96 samples derived by a previous study. They studied the methylation using three different three types of genetic analyzers for capillary electrophoresis in the following genes: *ELOVL2*, *FHL2*, *KLF14*, *MIR29B2CHG/C1orf132*, and *TRIM59* for blood and saliva and *TTC7B*, *LOC401324/cg12837463*, and *LOC729960/NOX4* specific for semen samples. The results showed a strong correlation, with a Pearson’s correlation index of 0.9, between predicted age and actual chronological age for blood and saliva samples. The index obtained from semen samples showed a moderate correlation. In the validation set the model gave an MAE value of 3.78 years for blood samples, 3.56 years for saliva, and, lastly, 4.55 years for semen samples. They also tested a combined model with both saliva and blood samples that obtained an MAE value of 3.94 years.

#### 3.7.7. Pyrosequencing

Although pyrosequencing could be considered a subtype of the NGS technique, in the field of human biological age prediction, it is often treated separately given its importance in age-related methylation analysis. Indeed, some scholars underlined how pyrosequencing represents a pivotal technique in this field due to its ability to precisely analyze DNA methylation levels at specific CpG sites, quantitatively controlling nucleotide incorporation through enzymatic conversion of pyrophosphate releasing a proportional light signal. Several studies have demonstrated the effectiveness of this approach on a wide range of biological sources, highlighting its versatility [9,71,142].

Numerous works have studied different genes from DNA extracted from blood samples.

Two hundred and forty-one bloodstain samples from Han Chinese people between the ages of 10 and 79 were examined in Yang et al.’s study [143]. The researchers created machine learning models, including an RFR model based on three CpG sites, using six CpG markers from genes like *ELOVL2*, *KLF14*, *TRIM59*, *C1orf132*, *FHL2*, and *NPTX2*. These models produced a MAD of 2.80 years for males and 2.93 years for females. The most reliable markers were found to be *TRIM59* and *C1orf132*.

Fan et al. [144] examined 240 peripheral blood samples from Southern Han Chinese individuals aged 1–81 years. They looked at 34 potential CpG sites, and because of their findings about the correlation with age, they focused on studying the methylation level of five genes. *TRIM59*, *FHL2*, *C1orf132*, *KLF14*, and *ELOVL2* were the five genes that were chosen. Out of all the machine learning models that were tested, the RFR model performed exceptionally well in younger people, with a MAD of 1.29 years for the whole dataset (MAD as low as 0.45 years in the 1–20 age group).

Park et al. [145] used a total of 2173 samples from a public database to identify AR-CpG sites, whereas 765 blood samples from healthy Korean volunteers were analyzed to predict chronological age. Data on several ethnic groups, including European, Dutch, Italian, Chinese, African American, and Caucasian populations, are included in these datasets. The study obtained a MAD of 3.16 years in the training set and 3.35 years in the validation set by combining three CpG sites, *ELOVL2*, *ZNF423*, and *CCDC102B*. These sites are beneficial in forensic applications such as crime scene reconstruction for age prediction. Their findings highlight how methylation profiles are consistent throughout many groups.

Ji et al. [146] concentrated on Y chromosomal markers unique to men. The strongest predictors in their analysis of 87 blood samples from 84 males and 3 females between the ages of 18 and 60 were found to be CpG3 (*cg27443332*) and CpG4. With a MAD of 5.50 years in the training set and 6.74 years in the testing set, the SVM model outperformed the other models examined. Subsequently, additional samples of different biological natures were used to evaluate the potential of the model in male–female mixtures. These analyses revealed that the age prediction model developed could be applied to the detection of male–female mixtures, even in cases where the male–female DNA ratio was as high as 1:50.

Huang et al. [147] created a regression model based on markers like *ITGA2B* and *NPTX2* after analyzing blood samples from 89 Chinese people, 42 of whom were female and 47 of whom were male and whose age ranged from 9 to 75 years. To validate the model, they also used 40 blood samples from participants ranging in age from 11–70. The methylation levels of the chosen CpG sites did not differ significantly by sex, and the combined dataset model obtained a MAD of 7.87 years. With a MAD of 7.99 years, they were able to attain forecast accuracy in the validation. Additionally, when applied to six bloodstains, that had been held for up to four months, the study showed consistent accuracy.

A total of 190 peripheral blood samples from patients with Graves’ disease and Alzheimer’s disease (early-onset and late-onset) were examined by Spólnicka et al. [148]. Furthermore, DNA methylation analysis was performed on 425 samples from an earlier study (305 training samples and 120 healthy controls). A multivariate regression model based on five genes, *ELOVL2*, *C1orf132*, *KLF14*, *TRIM59*, and *FHL2*, was created by their investigation. In younger early-onset Alzheimer’s disease patients, rapid hypermethylation on *TRIM59* resulted in a lower age prediction accuracy, with an MAE of 12.2 years, as opposed to 5.7 years for healthy controls.

The first study by Zbieć-Piekarska et al. [149] examined 427 blood samples from people between the ages of 18 and 75 as well as another group of samples from children between the ages of 2 and 17. The study concentrated on markers including *ELOVL2*, *C1orf132*, *TRIM59*, *KLF14*, and *FHL2*. The Standard Error was 4.5 years and the model’s MAD values were 3.4 and 3.9 in training and test sets, respectively. Moreover, a particularly effective model was that of the younger individuals, which achieved a MAD of 2.7 years in the training set and 2.8 years in the test set.

In another study, Zbieć-Piekarska et al. [150] focused on CpG sites in *ELOVL2*. They employed 303 samples in total to create a linear regression model that predicted age. A MAD of 5.03 years was determined from the data analysis. With a MAD of 5.75 years, this model showed strong performance in validation datasets of 124 samples that produced reliable findings.

Thong et al. [151] applied ANN to analyze 333 samples from a Singaporean, Malaysian, and Indian cohort aged from 0 to 88 years. The DNA methylation of four age-associated genes, *TRIM59*, *KLF14*, *FHL2*, and *ELOVL2*, was examined in this work. With a MAD of 3.7 years, the ANN model demonstrated exceptional accuracy, while *ELOVL2* showed the strongest connection. The model’s performance was barely affected by ethnic heterogeneity in this study.

Smeers et al. [152] analyzed 206 samples from individuals aged 0 to 91, focusing on four genes (*ELOVL2*, *EDARADD*, *PDE4C*, and *ASPA*). Their quantile regression model yielded consistent MAD values of about 3.26 years.

Feng et al. [153] examined a training set of 390 blood samples from Han Chinese males aged from 15 to 75 years, and then a pool of three test sets of samples (192 in total). Their multivariate regression model achieved MAD values between 2.71 and 2.91 years in the training set and between 2.47 and 4.41 years in validation sets, always considering the nine-CpG site model.

Fleckhaus and colleagues [154] analyzed 204 samples in order to compare populations in the Middle East and Central Europe to study the correlation of the DNA methylation level and chronological age in *ELOVL2*, *MIR29B2CHG*, *FHL2*, *KLF14*, and *TRIM59* genes. The need for population-specific modifications is shown by the fact that population-specific models produced MADs of 2.72 years for Central Europe and 3.34 years for the Middle East.

Using five genes (*ELOVL2*, *TRIM59*, *C1orf132*, *KLF14*, and *FHL2*), Anaya et al. [155] have examined 264 postmortem samples between ages 0 and 93, identifying *TRIM59* and *ELOVL2* as key markers. In the test set of 72 samples, their model achieved an average age of 7.42 years, with low accuracy for older people.

Cho et al. [50] used previously validated models on Korean individuals. Analyses performed on 100 samples aged between 20 and 74 years achieved a MAD value of 4.18 years. Accuracy was improved combining methylation and sjTRECs, considering model 4 obtaining a MAD value of 3.29 years.

Xiao et al. [156] found 785 age-associated CpGs in 350 samples of Han Chinese participants. Stressing the importance of customized predictive models, their gender-specific models reached MAEs of 2.79 years for males, 2.53 years for females, and 3.3 years considering both sexes together.

Kampmann et al. [157] conducted a cross-validation on 49 blood samples of people aged 18–64 years, evaluating the methylation levels of five genes: *ELOVL2*, *FHL2*, *KLF14*, *TRIM59*, and *C1orf132*. This study tested the validity of a multilocus regression model using blood samples from five different laboratories. Compared to loci such as *ELOVL2*, *KLF14*, and *FHL2*, which showed a strong correlation with age, *C1orf132* showed less satisfactory results. The model showed good performance in the 20–29 age group and had a MAD value of 3.62 considering all 49 samples. Markers such as *ELOVL2* and *KLF14* showed good reproducibility despite different laboratories.

Finally, Sukawutthiya et al. [158] analyzed 136 samples (100 fresh and 36 postmortem). Using CpG sites in *ELOVL2*, their model achieved a MAD of 4.2 years for individuals aged 21–60 years, with high specificity and precision.

Other works have studied the methylation level of genes from DNA extracted from buccal and saliva samples.

In the work of Guan et al. [159], 461 buccal swabs from the Han Chinese population were analyzed. The authors found strong correlations between methylation levels and age by looking for CpG sites in the *ELOVL2*, *TRIM59*, and *HOXC4* genes. With a correlation index of 0.71, *HOXC4* showed the highest correlation. The random forest model obtained more accurate predictions for the age group of 30 to 40 years with an MAE of 2.12 years in the training set and 4.39 years in the test set.

Shiga et al. [160] examined 102 buccal cell samples from healthy Japanese volunteers aged 21–77 years. They focused on twelve CpG sites in *TRIM59*, *PDE4C*, *ELOVL2*, and an intergenic region. A regression model showed a strong correlation between methylation levels and age, with a correlation index of 0.86 and a MAD value of 3.88. The value was slightly lower for the 20–40 age group and higher for the over 50s age group.

Koop et al. [161] examined the effect of decomposition on DNA methylation, working on two independent validation sets of 71 living individuals and 52 postmortem samples. In both sets of buccal swabs, a strong correlation was found between methylation at the CpG sites of *PDE4C*. MAD was 7.8 years for the living samples and 9.1 years for the postmortem samples. Decomposition had no effect on the methylation-dependent age estimate, as methylation levels remained stable at all stages of decomposition.

Mayer et al. [162] analyzed 199 buccal mucosa samples from 104 children with growth disorders and 95 healthy children (age 0.42–18 years). The authors studied 22 CpG sites and discovered 11 CpGs that showed strong correlations with age. With an MAE of 2.21 years, the models based on healthy children overestimated age in the groups with growth disorders. CpGs in *RPA2*, *ELOVL2*, and *PDE4C* showed the most notable differences. Moreover, an MAE of 1.79 years was associated with healthy individuals.

Becker et al. [163] observed similar methylation patterns between populations by comparing saliva samples from 368 German and 89 Japanese donors. With correlation index values around 0.95, the *ELOVL2* and *PDE4C* genes showed the strongest correlations with age. In both groups, the prediction models obtained similar MAEs: the Germans obtained an MAE value of 4.14 years, while in the Japanese population an MAE value of 4.38 years was obtained.

In the study by Siahaan et al. [164], samples from 90 patients who underwent oral surgery were examined for DNA methylation in genes such as *PDE4C*, *RPA2*, and *ELOVL2*, as well as for morphological markers such as dental mineralization. Analyzing the level of methylation of 88 buccal swabs, the model achieved the highest accuracy, with an MAE value of 3.85 years, for the individuals under 28 years. For the overall dataset an MAE of 4.14 years was obtained considering only DNA methylation. A combined model achieved the highest accuracy with an MAE of 2.81 years for individuals under 28 years, considering dental mineralization in addition to DNA methylation markers.

Studies were also conducted on several biological matrices to assess the possibility of conformation among them.

In the work of Pfeifer et al. [165], 151 blood samples and 149 buccal swabs from deceased and living individuals, respectively, were used. Moreover, another 21 blood samples from living individuals were collected. All these samples were analyzed to study methylation in the *EDARADD*, *ASPA*, *PDE4C*, and *ELOVL2* genes. Without significant differences between fresh and stored samples or between living and deceased, the results showed strong correlations between methylation levels and age. The MAD values were 9.84 in blood samples and 8.32 years in buccal swab samples.

In another significant study, Alghanim et al. [166] examined methylation at the *SCGN*, *DLX5*, and *KLF14* loci with 72 blood samples from volunteers aged 5–72 years and 91 saliva samples from individuals aged 6–73 years. The single locus model of saliva had a MAD value of 5.8 years in the training set and of 8 years in the validation set. Differently, considering blood samples, MAD values of 6.6 years and 10.3 years were obtained in training and test sets, respectively. These results show that saliva has a higher specificity for estimating age than blood, underlining how important the tissue matrix is for creating predictive models.

Bekaert et al. [167] also conducted an in-depth analysis of blood samples. They examined 206 blood samples in total and 29 dental samples, focusing on the *ASPA*, *ITGA2B*, *EDARADD*, *ELOVL2*, and *PDE4C* genes. The most accurate marker among these was CpG6 of *ELOVL2*, which resulted in a MAD of 3.75 in blood and of 4.86 in teeth. However, accuracy decreased in subjects over 60 years of age, indicating that more age-appropriate models are needed.

Studies have also been conducted analyzing sperm samples as a biological source, which is an interesting matrix and certainly of interest in the forensic field. Finally, studies were carried out on biological sources such as nails and teeth.

Li et al. [168] analyzed a total of 92 samples of different biological origins, focusing on semen. In this context they found two age-related CpGs in sperm (*cg06979108* and *cg12837463*) and strong correlation indexes with MAD values of approximately 4 years.

An additional investigation was conducted by the team of Soares Bispo Santos Silva et al. [169] who examined particular markers such as *GRIA2* and *NPTX2* in 23 blood and 44 saliva samples. The results showed that *GRIA2* provided greater accuracy in saliva than in blood with a correlation index of 0.80 versus 0.55. The *NPTX2* gene maintained a similar correlation between the matrices but provided less accurate predictions. Over the growth period, the *GRIA2* gene obtained a MAD value of 6.9 years, while the *NPTX2* gene obtained a MAD value of 9.2 years. This work highlights how the predictive capabilities of models can be influenced by tissue-specific differences.

Fokias et al. [170] examined the methylation of 15 CpG sites in the *ASPA*, *EDARADD*, *PDE4C*, and *ELOVL2* genes in 108 living volunteers aged 0–96 years in nail samples and 5 deceased individuals. The results showed a strong correlation between methylation levels and age, with significant differences in methylation patterns between toenails and fingernails. A predictive model that was developed obtained a MAD of 4.76 years in the training set and 5.48 years in the test set. Postmortem samples also gave accurate predictions in most cases, confirming that nails are a reliable forensic matrix.

In a follow-up study, Fokias et al. [171] analyzed 91 sampled collected from 86 living individuals and 5 cadavers, focusing their attention on other markers, such as *TRIM59*, *MIR29B2CHG*, and *KLF14*, to improve the estimation models of fingernails and toenails. With a correlation index of approximately 0.808, the methylation levels in *TRIM59* showed the most significant correlations with age. Compared to 8.42 years in the original model, the MAD of the new models was reduced to 5.61 years in the test set. The predictions for the postmortem samples showed a significant improvement, with the MAD reduced from 11.76 to 3.90 years, demonstrating the effectiveness of the updated models.

Weidner et al. [172] observed a most accurate age prediction using DNA methylation rather than telomere length, obtaining a MAD of 5.4 years for the training set and 4.5 years in the test set starting from 151 blood samples.

Another study focusing on both DNA methylation and telomere length analysis, but using teeth, was that of Márquez-Ruiz et al. [173]. They examined the methylation of 21 CpG sites in the *ELOVL2*, *PDE4C*, and *ASPA* genes, together with the relative length of telomeres in 65 samples of individuals aged between 15 and 85 years. The most significant association between age and CpG sites in *ELOVL2* was shown. Multivariate quantile regression models were developed using data on telomere length and DNA methylation. Two model sets were developed: one excluded the variable sex, the other included the variable sex. Several models were developed and tested for their MAE. A major model using nine CpG sites from *ELOVL2* and *PDE4C* produced an MAE of 5.08. Accuracy was highest in the younger age groups and gradually decreased with age.

Finally, Zapico et al. [174] examined the methylation of 46 CpG sites in five genes: *KLF14*, *NPTX2*, *ELOVL2*, *FHL2*, and *SCGN*. They examined 20 dental pulp samples. The most accurate model, based on selected CpGs, achieved an MAE of 1.55 years, demonstrating a very accurate age estimation. The developed models showed a strong correlation between chronological and predicted age, even with a limited sample size. This underlines the possibility that dental pulp can be used for forensic applications.

#### 3.7.8. Other Techniques for DNA Methylation Analysis

Nanopore sequencing and adapting sampling techniques used for enrichment of regions of interest are certainly among the most innovative approaches, as the study conducted by Yuen et al. [175] including both genetic markers related to age estimation and markers for biological fluid identification shows. This study considered a pool of 10 blood samples, with individuals aged between 25 and 76 years. Reference DNA samples with methylation values were also analyzed. This study emphasized that the *ELOVL2* gene (*chr6:11044644*) has a very strong correlation with the prediction of chronological age, obtaining a Pearson correlation value of r = 0.948, confirming what has been reported in the literature. In the same study, additional age-associated markers such as *LINC02766* were also analyzed; this nanopore sequencing method showed similar efficacy compared to traditional standard sequencing techniques, obtaining a Pearson’s correlation index of r = 0.86. This result highlights that it is an optimal technique for age estimation and for body fluid identification analysis simultaneously.

Another study conducted on nanopore sequencing was conducted by de Bruin and colleagues [176]. In their study they compared the performance of Oxford Nanopore Technologies, third-generation sequencing, to standard MPS data and Illumina EPIC DNA methylation profiles. Samples of six males aged 19–53 years were used, analyzing a total of 361 markers obtained from previous studies. The obtained results revealed a positive correlation between age and *FHL2*, *TRIM59*, *ELOVL2*, and *PDE4C* and a negative correlation with *MIR29B2CHG*. Differently from other studies, *ELOVL2* showed a very low Pearson’s correlation coefficient, suggesting that further research is needed to understand the differing results observed.

Noroozi et al. [177] analyzed a total of 962 samples from blood and buccal swabs using epigenetic clock models such as Skin&Blood, GrimAge, and PhenoAge, in combination with different normalization techniques and Illumina microarrays. The results showed that the Skin&Blood clock was the most accurate for blood samples, with an MAE value of 2.47 years, while for buccal swabs the data were less accurate with an MAE value of 3.86 years. The analysis also explored how the impact of genetic and lifestyle factors is reflected in a slowing down of epigenetic aging by avoiding bad habits, such as excessive consumption of coffee, meat, or alcohol, smoking, or little exercise.

Droplet digital PCR (ddPCR) is a further technique used that is able to obtain an accurate quantification of DNA methylation. Manco and Dias [178] conducted a study using ddPCR as the main technique and analyzed 56 peripheral blood samples from healthy individuals aged between 1 and 94 years. The model developed is based on the study of five CpG sites of the *ELOVL2* gene. The methylation level and the chronological age showed a significant correlation with a correlation index of R^2^ = 0.792, while the MAD underlined an age range of approximately 10 years, which was also confirmed by cross-validation tests. The lowest accuracy was found in the older age category. In another study, the same authors [179] explored the combination of three different genes (*ELOVL2*, *FHL2*, and *PDE4C*) to assess their improvement from a predictive point of view of the final age range. The study analyzed 58 peripheral blood samples donated by healthy individuals aged between 1 and 93 years of Portuguese origin. The combination of these three markers resulted in a statistically significant correlation index of R^2^ = 0.937. They then performed tests on a combined model, validated by cross-validation, which resulted in a MAD of 4.66, which is much better than the single gene tests. In their study, the most reliable gene was *FHL2* with a MAD of 6.03 years, followed by *ELOVL2* (MAD = 7.30 years) and *PDE4C* (MAD = 10.85 years).

Ho Lee et al. [180] also used ddPCR as a technique for methylation analysis, focusing on four genes (*SST*, *KLF14*, *TSSK6*, and *SLC12A5*). The salivary samples used were taken from 76 volunteers of different ages. The results obtained from this study showed that there is a very strong correlation between methylation levels and the chronological age of individuals in multiple regression models. The model of methylation levels performed better in younger individuals, e.g., for the age group of around 20 years (MAD of approximately 2.57 years) while for the group of 50-year-olds, the MAD increases to a value of 4.35 years. The predictive accuracy was further analyzed and validated by means of the leave-one-out cross-validation (LOOCV) model, obtaining a MAD of 3.3 years despite the limited sample size.

A different technique for studying methylation levels uses the EpiTect Methyl qPCR Assay which does not require bisulfite conversion for the study of methylation patterns. Using blood samples from 80 females between the ages of 18 and 91, Mawlood et al. [181] investigated 13 genes (*NPTX2*, *KCNQ1DN*, *GRIA2*, *TRIM58*, *EDARADD*, *FGF7*, *ZC3H12D*, *TOM1L1*, *SOGA1*, *BCAS4*, *ASPA*, *PDE4C*, and *ITGA2B*). The quadratic correlation (R2) values for four genes (*NPTX2*, *GRIA2*, *TRIM58*, and *KCNQ1DN*) ranged from 0.452 to 0.808, indicating a substantial association with age. The MAD value for the entire prediction model is approximately 11 years, which is significantly higher than the MAD value of 7.2 years obtained from the model that evaluated the methylation level of the four best-performing genes.

Recently, using double-enzyme reduced representation bisulfite sequencing (dRRBS), Li and colleagues [182] published a study based on the genome-wide identification of AR-CpG markers in semen. This was followed by a reliable two-stage validation technique for age detection. They used a cohort of semen samples supplied by 21 people in three age groups (young, middle-aged, and older) and 261 samples for validation. With an average MAE of 3.30 years, the final age estimation model, which was based on a random forest algorithm and used a refined set of nine CpG sites, showed strong performance.

Qian et al. [183] screened for 811,876 CpGs from whole blood of 3312 Chinese individuals ranging from 18 to 83 years of age based on a stepwise conditional epigenome-wide association study (SCEWAS). Forensic applications could benefit greatly from their systematic genome-wide feature selection, which was limited to a narrow panel of 10 CpGs for precise age prediction. With a MAD of 3.20 years, the model’s predictions were extremely accurate in a test group of Chinese people (*n* = 648). As may be predicted given varied genetic backgrounds, the model was further tested in a publicly available dataset of multiple ancestral origins (86 Europeans, 14 Asians, and 273 Africans). The data revealed a different MAD of 6.21 years.

#### 3.7.9. Combined Techniques

Different studies in the literature reported the use of more than one technique for age determination in the forensic field, including at least one based on DNA methylation.

By analyzing the CpG sites of *ELOVL2*, *FHL2*, and *MIR29B2* in 84 blood samples from individuals aged 18 to 99, Freire-Aradas et al. [184] compared several age prediction models, obtaining different MAE values, using data from four DNA methylation technologies: MassARRAY (EpiTYPER), pyrosequencing, NGS (miniSeq), and SNaPshot. They discovered that the methylation patterns of the CpG sites for *ELOVL2* and *FHL2* were similar across pyrosequencing, MiSeq, and EpiTYPER, which means that data from these methods can be used for age prediction models that are independent of the platforms to be used. *MIR292B*, on the other hand, consistently showed non-uniformity and went beyond the bound of agreement. Furthermore, if the sample size differs from the training set of the original model, utilizing platform-independent models may result in lower prediction accuracy. This challenge might be resolved by expanding the sample sizes evaluated across technologies or by reexamining training sets using the relevant technology.

In the study of Shi et al. [185], it was shown that using age-associated DNA methylation markers in conjunction with conventional techniques for skeletal age and dental age (SA and DA) greatly increased the precision of locating age-associated CpG sites found in the genes *DDO*, *PRPH2*, *DHX8*, and *ITGA2B*. The MAE dropped to 0.47 years for males and 0.34 years for girls when age-associated DNA methylation markers were included for SA and DA in the age estimation model. They used X-ray inspection to measure SA (GP, TW3-RUS, and TW3-Carpal techniques) and DA (Demirjian and Willems methods) and NGS in parallel with ddPCR in order to validate age-associated CpG sites in 124 Chinese children (78 males and 46 females) between the ages of 6 and 15.

The study conducted by the research group of Xu et al. [186] used blood samples to identify CpG age-associated sites to develop a predictive model to estimate chronological age through the level of DNA methylation. They studied biological samples from 8 pairs of female monozygotic twins, aged 21–32 years, and 50 healthy unrelated volunteers, without acute or chronic diseases, aged 20–80 years, with Han Chinese origin. The NGS approach was used to assess the DNA methylation, measuring approximately 485,000 CpG sites, whereas a MassARRAY platform was used to validate particular sites. As a result, 2965 sites were found to be associated with age, and a positive regression was observed at the level of 1476 sites and a negative regression was observed in a total of 1489 sites. A more thorough examination revealed 11 locations that were strongly linked to age. The study also found novel genes that may be implicated in aging, such as *ADAR*, *AQP11*, *ITGA2B*, and *PDE4C*. Numerous statistical and machine learning algorithms have been created to predict chronological age. The MAD of 2 years in the original dataset, using support vector regression model, was the most accurate; however, in the validation set, the MAD value increased to about 6 years.

Montesanto et al. [187] conducted an extensive study of different genes using two different techniques. Using the MassARRAY technique, they quantified the DNA methylation level in the following genes: *C1orf132*, *KLF14*, *TRIM59*, and *FHL2*. On the other hand, with the pyrosequencing technique they focused on nine CpG sites in the *ELOVL2* gene. Peripheral blood samples were analyzed from 330 unrelated individuals, divided into 146 men and 184 women, aged between 20 and 100 years. Eight CpG markers were selected and several sites associated with age were identified, with a greater presence in the genes *ELOVL2*, *C1orf132*, *TRIM59*, and *FHL2*. With an MAE of 5.13, the predicted and chronological ages showed a strong correlation. The model was then validated on a test set consisting of samples donated by a total of 83 individuals, obtaining an improved MAE with a value of 4.5 years. However, even during the validation phase, the data obtained showed a decrease in precision with increasing age.

Lastly, Schwender et al. [188] analyzed 141 salivary samples from 94 females and 47 males aged between 21 and 69 years. The analysis conducted assessed the methylation level of 54 CpG sites at the level of six genes, *PDE4C*, *ELOVL2*, *EDARADD*, *SST*, *KLF14*, and *SLC12A5*, by means of two different techniques: pyrosequencing and minisequencing, with the aim of assessing their differences. The results showed a negative correlation for the CpG sites of *EDARADD*, while other genes showed positive correlations with age. Prediction models were developed based on three CpG sites that showed a high accuracy for age estimation. The data obtained by pyrosequencing showed a strong level of correlation and obtained a MAD value of 5.11 years for the training set. The result was confirmed by using an independent set that obtained a MAD value of 5.33 years. Similarly, the model based on the minisequencing technique obtained a MAD value of 5.16 years for the training set and 6.44 years for the validation set. Both models showed a better performance in the female sample pool than in the male sample pool. Furthermore, methodological observations showed greater manual variability in minisequencing than in pyrosequencing, which partially explains the lower accuracy of the latter. Nevertheless, both methods produced robust results, which support the applicability of the models for chronological age estimation in forensics.

## 4. Discussion

Age estimation methods based on biochemical processes have gained prominence in forensic science due to their ability to minimize variability introduced by individual biological differences. The techniques analyzed, including radiocarbon dating, aspartic acid racemization (AAR), and signal joint T-cell receptor excision circles (sjTRECs) quantification, have demonstrated varied accuracy and applicability depending on the biological matrix and context of application. The diversity of methods for age estimation highlights distinct advantages and trade-offs among techniques. By analyzing these differences, we can better understand their practical applications, limitations, and synergistic potential when combined.

Radiocarbon dating, leveraging the “bomb pulse” effect, is arguably the most precise method for estimating birth years, particularly for individuals born during or after the mid-20th century. Its accuracy is unparalleled, with minimal errors demonstrated across various studies. However, its applicability is limited by high costs, labor-intensive protocols, and diminished precision for individuals born before 1955. Radiocarbon dating stands out for its precision in estimating the year of birth based on the incorporation of atmospheric 14C during tissue formation. Studies such as those by Alkass et al. [18] and Teglind et al. [17] have illustrated that enamel’s fixed radiocarbon levels, aligned with atmospheric calibration curves, provide birth year estimates with errors often below 1.5 years. However, the method’s reliance on the bomb pulse period (1955–1963) limits its applicability to individuals born outside this timeframe. The requirement for accelerator mass spectrometry (AMS) introduces significant costs and complexity, reducing its accessibility for routine forensic use. Furthermore, while enamel proves resilient to environmental contamination, bone turnover rates challenge its utility in postmortem contexts.

The integration of radiocarbon dating with methods like AAR offers a complementary approach, addressing some limitations of each technique. For example, while radiocarbon dating provides an estimated birth year, AAR can determine the individual’s chronological age at death, yielding a comprehensive forensic profile.

AAR has long been recognized for its ability to estimate age through the predictable accumulation of D-aspartic acid in stable proteins. AAR offers a universal, protein-based approach to age estimation by analyzing the predictable racemization of aspartic acid in stable proteins. Studies have validated its use in dentin, cartilage, and sclera, highlighting a strong correlation with chronological age and its potential flexibility in forensic contexts. Studies across populations consistently show strong correlations between D/L ratios and chronological age, with precision enhanced by methods like HPLC. For instance, Sakuma et al. [23] achieved a correlation coefficient of 0.98 in dentin, underscoring the method’s reliability. However, variability arising from protein composition, environmental conditions, and sample handling underscores the need for careful calibration and standardization. Despite its destructive nature and sensitivity to external factors, AAR complements radiocarbon dating when used in tandem, offering dual insights into chronological and biological age. However, AAR is not without limitations. Factors such as caries, environmental conditions, and protein composition introduce variability. While alternative tissues like sclera and cartilage expand its applicability, they exhibit lower correlations compared to dentin. Heat-treatment techniques, as developed by Minegishi et al. [32], offer innovative solutions to accelerate racemization and improve reproducibility, but further validation is needed to establish these methods as forensic standards.

The application of mtDNA biomarkers is based on the accumulation of somatic mutations and deletions over time, offering robust correlations with age. mtDNA’s susceptibility to oxidative damage makes it a promising marker for age estimation. Research by Zapico et al. [38] and Lacan et al. [39] has confirmed age-related accumulation of mtDNA deletions and mutations in high-energy tissues, offering a molecular clock for forensic applications. However, the heterogeneity of mtDNA damage across tissues and its influence by oxidative stress limit its universality. Future work should address these variations and explore its integration with other biomarkers. Their susceptibility to oxidative damage makes them particularly relevant in high-energy tissues like skeletal muscle and heart. While mtDNA’s stability and the feasibility of using degraded samples provide advantages, variability in mutation accumulation across individuals poses a challenge. Population-specific calibration and standardized protocols are essential to harness the full potential of mtDNA in forensic applications.

sjTRECs quantification is another highly promising method for age estimation, with strong correlations demonstrated across diverse populations. Its reliance on the predictable decline of thymic output with age ensures biological universality. Quantitative PCR-based studies, such as those by Ou et al. [42,47] and Cho et al. [50], demonstrated a strong negative correlation between sjTREC levels and chronological age, with error margins ranging from ±7 to ±10 years. The method’s adaptability to dried bloodstains extends its forensic utility, though sample degradation and health-related immune senescence pose challenges. Notably, integrating sjTRECs quantification with DNA methylation or telomere length has yielded enhanced predictive models, emphasizing the value of multimodal approaches, as demonstrated by Elmadawy et al. [51]. However, factors such as health conditions and immune system variability can weaken the correlation. This integrative approach should be a key focus of future studies. The limitations of sjTRECs in older and degraded blood samples, as well as their sensitivity to environmental factors, highlight the need for robust storage and handling protocols. Additionally, the method’s applicability to forensic scenarios involving dried bloodstains is a notable advancement, although challenges in standardization still remain.

Advancements in transcriptomics have highlighted the forensic potential of RNA markers, particularly non-coding RNAs (ncRNAs) such as miRNAs, circRNAs, and piRNAs, which have emerged as powerful tools for forensic age estimation. The dynamic and tissue-specific nature of RNA makes it ideal for determining both sample origin and chronological age, even in degraded samples. These markers exhibit tissue specificity and stability, enabling their use in degraded samples. Wang et al.’s studies [63,64] on miRNAs and circRNAs have shown MAEs as low as 3.68 years, particularly when combined in machine learning models. However, variability introduced by environmental influences and limited sample sizes necessitates further validation and standardization. Notably, studies employing mRNA demonstrated its potential as an independent and complementary biomarker to DNA methylation markers. However, mRNA alone has lower predictive accuracy compared to DNA methylation, which limits its standalone application. Instead, its integration into multimarker models, as shown by Zubakov et al. [59], enhances precision. miRNA and piRNA offer advantages over other RNAs due to their stability and tissue specificity. Their role in machine-learning-driven predictive models, as illustrated by Fang et al. [61], is promising. However, challenges such as sex-specific prediction errors, lower accuracy in females, and the absence of pediatric samples indicate the need for more inclusive research to refine these methodologies. circRNAs, although less studied, have shown potential in age estimation, particularly through machine learning models. Their role in regulating gene expression through miRNA interactions is a novel avenue for forensic research. However, the higher error margins and limited sample sizes indicate that further exploration is needed to validate their utility across diverse populations and age ranges.

DNA methylation profiling has revolutionized human chronological forensic age estimation, with CpG sites like *ELOVL2* consistently emerging as key predictors. Techniques ranging from Sanger sequencing to next-generation sequencing (NGS) have enabled precise modeling across various biological matrices. Freire-Aradas et al. [90] demonstrated MAEs below 4 years using NGS, while Marcante et al. [130] achieved 3.49 years in saliva-specific models. The VISAGE project further highlighted the robustness of methylation-based methods across tissues and populations. However, variability in methylation rates due to health, ethnicity, and environmental factors underscores the need for tailored calibration models. The integration of multiple biomarkers—sjTRECs, DNA methylation, AAR, and others—offers a promising avenue to overcome individual method limitations. Studies by Elmadawy et al. [51] and Cho et al. [50] have shown that combining biomarkers significantly improves accuracy and robustness. This multimodal approach aligns with the complex nature of aging, which is influenced by genetic, epigenetic, and environmental factors.

While significant strides have been made, several challenges remain. The destructive nature of some analyses, high costs, and the need for sophisticated equipment limit widespread application. Furthermore, population-specific calibration remains a critical consideration, as demonstrated by differences in methylation patterns across ethnic groups. Future research should focus on standardizing methodologies, expanding sample diversity, and leveraging machine learning to optimize predictive models. Additionally, exploring novel biomarkers and enhancing multimodal integration will further refine forensic age estimation.

## 5. Conclusions

When comparing the various age estimation techniques, each offers distinct advantages and limitations. Radiocarbon dating, while highly precise for individuals born during the bomb pulse era, is constrained by its reliance on specific historical atmospheric conditions. Its application is also limited by high costs and the specialized equipment required, such as AMS. In contrast, AAR provides a broader temporal applicability and can analyze multiple tissue types. However, its susceptibility to environmental factors and the destructive nature of the method pose significant challenges.

mtDNA analysis offers the advantage of a molecular clock based on oxidative damage, making it particularly useful in high-energy tissues like muscles and the brain. Nevertheless, the variability in mtDNA mutation rates and the influence of oxidative stress limit its generalizability. Similarly, sjTRECs quantification benefits from its correlation with thymic output, providing insights into immune aging. However, its precision diminishes with advanced age, and health conditions can skew results.

RNA-based methods represent a newer frontier, leveraging the stability and tissue specificity of ncRNAs. These markers, such as miRNAs and circRNAs, have shown great promise in forensic applications due to their ability to remain stable even in degraded samples. Despite this, the field is still developing, and further research is needed to address the variability introduced by environmental factors and limited dataset sizes.

DNA methylation stands out as one of the most versatile and widely studied methods. The analysis of CpG sites, particularly through advanced techniques like NGS, provides highly accurate age predictions across a variety of biological matrices. The integration of DNA methylation with other markers, such as sjTRECs or RNA profiles, has further enhanced predictive accuracy. However, the technique’s sensitivity to external factors like health and ethnicity underscores the need for robust calibration models.

This comparative analysis illustrates that no single method is universally superior; instead, their strengths and limitations should guide their application based on the forensic context. The greatest potential lies in integrating these techniques into multimarker models, supported by advances in machine learning, to achieve unparalleled accuracy and reliability in age estimation.

The methods reviewed demonstrate the growing sophistication of molecular age estimation in forensic science. By leveraging biochemical processes, researchers have achieved unprecedented precision and reliability. However, addressing methodological and contextual limitations will be essential to maximize their potential. As the field advances, the integration of complementary biomarkers and innovative technologies promises to redefine forensic human chronological age estimation, enhancing its applicability across diverse scenarios.

Future studies should also emphasize standardization, particularly in sample handling and analysis protocols, to ensure reproducibility and reliability across forensic contexts. By leveraging the complementary strengths of these methods, forensic scientists can build robust, accurate models for age estimation, advancing the field toward greater precision and applicability.

## Data Availability

The summary of data presented in this study is attached as Supplementary Materials.

## Supplementary Materials

The following supporting information can be downloaded at: https://www.mdpi.com/article/10.3390/ijms26073158/s1.
