# Supplementary material for: Advancing Forensic Human Chronological Age Estimation: Biochemical, Genetic, and Epigenetic Approaches from the Last 15 Years: A Systematic Review
Source: Int J Mol Sci. 2025 Mar 28;26(7):3158. doi: 10.3390/ijms26073158 (PMC11988829; doi:10.3390/ijms26073158)
Supplement: Supplementary file 1 [file ijms-26-03158-s001.zip › ijms-3496375-supplementary.pdf]

Supplementary material

Table S1. Summarized table of study based on age-prediction based on Radiocarbon dating, Aspartic Acid Racemization, Mitochondrial DNA analysis, Signal Joint T-cell Receptor Excision Circles, RNA analysis, and Telomere Length analysis.

Article	*Biological Material*	*Samples Information*	*Main Results*
Radiocarbon dating			
Kondo-Nakamura et al. [15]	Teeth	8 samples from 5 individuals (4 males and 1 female – 29 to 75 years)	Deviation between estimated and real Date of Birth from - 2.72 to 2.70
Alkass et al. [16]	Teeth	95 samples from 84 individuals (17 to 100 years)	For tooth enamel laid down between 1955 and 1963 = 1.9 ± 1.4 years.<br>For tooth enamel laid down after 1963 = 1.3 ± 1.0 years.
Teglind et al. [17]	Teeth and bones	87 samples (63 teeth and 24 bones) from 52 individuals (15 males, 11 females, and 26 unkown sex)	Absolute error = 1.18 ± 0.83 years (using one tooth), 1.3 ± 1.2 (using two or more teeth).
Alkass et al. [18]	Teeth	48 samples of which 44 samples from 41 individuals (21 males and 20 females – 13 to 70 years) and 4 from one unsolved homicide	Absolute error = 1.0 ± 0.6 years, considering radiocarbon analysis<br>Absolute error = 5.4 ± 4.2 years, considering aspartic acid racemization
Aspartic Acid Racemization (AAR)			
Griffin et al. [22]	Teeth	129 samples, 31 of which from 31 modern individuals and 98 of which from three archaeological populations	Average variation = 7.1 years in modern individuals<br>Average variation up to 25 years considering individual from archaeological populations
Sakuma et al. [23]	Teeth	34 samples from 12 individuals for 24 samples (17 to 76 years) and 10 individuals (33 to 72 years)	r = 0.98 considering AAR related to chronological age for dentin<br>r = 0.93 considering AAR related to chronological age for whole tooth:<br>Difference from actual and estimated age between about – 0.12 and – 6.86 years
Sakuma et al. [24]	Teeth	8 samples from 8 individuals (17 to 61 years)	Differences between actual and estimated age in pink teeth between – 1 and + 3 years
Sirin et al. [25]	Teeth	102 samples, 99 of which from 23 individuals (21 to 79 years) and 3 control individuals (17 to 35 years)	Range between –1.87 and 3.69 years considering differences between estimated and real ages in control samples<br>Range between –12.67 and 20.39 years considering differences between estimated and real ages in affected samples
Arany et al. [26]	Teeth	24 samples from 24 individuals (13 to 88 years)	r ≈ 0.99 from the racemization rate of Aspartic Acid<br>r = 0.85 from the racemization rate of Glutamate<br>r ≈ 0.84 from the racemization rate of Alanine
Wochna et al. [27]	Teeth	75 samples from 16 male cadavers (20 to 68 years)	r = 0.96–0.98 considering correlation between age and AAR in teeth<br>SE = ± 2.95 to ± 4.84 years
Rastogi et al. [28]	Teeth	100 samples from 90 individuals (10 to 70 years), 10 of which underwent incorrect sampling/handling	Error in the range of 0 to ± 4 years
Chen et al. [29]	Teeth	58 samples from 58 individuals (24 males and 34 females – 18 to 63 years)	MAE = 2.19 years
Zapico et al. [30]	Teeth	20 samples from 20 individuals (12 males and 8 females – 19 to 70 years)	MAE = 5 years in training set<br>Difference in age estimation ± 2-6 years in test set

Elfawal et al. [31]	Teeth	89 samples from 89 individuals (59 males and 30 females – 10 to 31 years)	SEE = ± 1.26 years in test group<br>SEE = ± 1.12 years in validation group
Minegishi et al. [32]	Teeth	39 sampled from 4 individuals (25 to 28 years)	SEE = ± 5 years
Matzenauer et al. [33]	Cartilage	110 samples from 65 deceased individuals for cartilage (16 to 92 years) and 45 deceased individuals for purified elastin samples (24 to 96 years)	r = 0.76 considering AAR and age in cartilage samples<br>r = 0.84 considering AAR and age in purified elastin samples
Klumb et al. [34]	Eyes	75 samples from 75 individuals (6 to 100 years)	r = 0.92 considering ARR and age in training test<br>Age range deviation between ± 6.5 and ± 32.35 years in validation set
Mitochondrial DNA (mtDNA) analysis			
Zapico et al. [38]	Teeth	49 samples from 49 individuals (20 to 70 years)	Negative correlation between defective HV2 amplification events and age in dentin (p ≤ 0.01)
Lacan et al. [39]	Muscle and bone	164 samples, one of each biological material from 82 deceased individuals (54 males and 28 females – 2 to 87 years)	Duplication analysis revealed age-dependent patterns, with up to 3 duplications in muscle tissue and 2 in bone, predominantly in older individuals
Signal Joint T-cell Receptor Excision Circles (sjTRECs)			
Ou et al. [42]	Blood	284 samples from 284 individuals (145 males and 103 females – 0 to 78 years)	r ≈ - 0.8177<br>SE = ± 10.47 years
Cho et al. [43]	Blood	172 samples from 172 individuals (16 to 65 years)	r = - 0.807<br>SE = ± 8.49 years
Ibrahim et al. [44]	Blood	153 samples from 153 individuals (63 males and 90 females – 0 to- 70 years)	SE = ± 7.35 years
Zubakov et al. [45]	Blood	195 samples from 195 individuals (0 to 80 years)	SE = ± 8.9 years
Yamanoi et al. [46]	Blood	207 samples from 201 individuals (111 males and 90 females – 18 to 89 years) for 201 fresh samples and 6 dried spots	SE = ± 8.0 years
Ou et al. [47]	Blood	372 samples from 264 individuals (146 males and 118 females – 0 to 86 years) for both 264 fresh samples and 31 stored samples, and 77 stored older samples	r = - 0.871<br>SE = ± 9.42 years
Cho et al. [48]	Blood	321 samples from 149 diseased individuals (7 to 90 years) and 172 healthy individuals (16 to 65 years)	MAD ≈ 11.59 years
Farag et al. [49]	Blood	233 samples from 148 diseased individuals (67 males and 81 females – 0 to 80 years) and 85 healthy individuals (36 males and 49 females – 0 to 80 years)	MAD = 9.40 years for healthy individuals<br>MAD= 11.04 years for autoimmune patients<br>MAD = 9.71 years for COVID-19 patients
Cho et al. [50]	Blood	100 individuals (50 males and 50 females – 20 to 74 years)	MAD =10.33 years
Elmadawy et al. [51]	Blood	124 samples from 124 individuals (68 males and 56 females – 0 to 78 years)	SE = ± 12.6 years
RNA analysis			
Deng et al. [58]	Blood	150 samples from 150 individuals (73 males and 77 females – 0 to 83 years)	ERCC1 and XPF mRNA expression levels declined in an age-dependent manner (r = - 0.578 / - 0.844)
Zubakov et al. [59]	Blood	267 samples from 267 individuals (125 males and 142 females – 22 to 84 years)	MAD = 9.20 years
Dørum et al. [60]	Blood	352 samples from 117 healthy male individuals and 235 publicly available sequencing data	MAE = 4.35 years based on U.S. dataset<br>MAE = 7.78 years based on DESeq2 list
Fang et al. [61]	Blood	220 samples from 220 individuals (110 males and 110 females – 20 to 69 years)	MAE = 5.52 years in male<br>MAE = 7.46 years in female
Fang et al. [62]	Blood	240 sample from 240 males (20 to 69 years)	MAE ≈ 3.17 years in validation set
Wang et al. [63]	Blood	143 samples from 143 individuals (69 males and 74 females – 19 and 73 years)	MAE ≈ 8.77 years regression tree

			MAE ≈ 9.13 years random forest regression
Wang et al. [64]	Blood	419 samples from 200 individuals (98 males and 102 females – 20 to 80 years), 4 young individuals (20-29 years), 4 elderly individuals (50-62 years), 40 individuals (19-73 years, and 171 from public datasets	MAE = 3.68 years in training set<br>MAE = 6.84 years in test set
Salignon et al. [66]	Blood	103 samples from 103 individuals (91 males and 12 females – 20 to 83 years)	R^2 proteins = 0.59<br>R^2 miRNAs = 0.54<br>R^2 combined =0.70
Telomere Length analysis			
Elmadawy et al. [51]	Buccal swabs	248 samples from 124 individuals (68 males and 56 females – 0 to 78 years)	Mean prediction error = 12.5 years
Srettabunjong et al. [67]	Blood	100 samples from 100 individuals (50 males and 50 females)	Quadratic correlation index with a value of 0.391
Magi et al. [68]	Muscle biopsies	26 samples from 26 males (20 to 50 years)	
Márquez-Ruiz et al. [69]	Teeth	91 teeth from 77 individuals (20 men and 53 woman – 15 to 85 years)	MAE ≈ 9.85 years
Tejasvi et al. [70]	Dental pulp	30 samples from 30 individuals (15 to 35 years)	Kilobase-level reduction ranging from 9.92 for young individua to 9.13 for older ones

Table S2. Summarized table of study based on age-prediction based on DNA Methylation Analysis.

Article	*Biological Material*	*Samples Information*	*Methodology Applied*	*Main Results*
DNA Methylation – Sanger sequencing				
Correia Dias et al. [75]	Blood	70 samples of deceased individuals	Bisulphite conversion	MAD = 6.08 years in training set<br>MAD = 8.84 years in test set
Correia Dias et al. [76]	Blood	71 samples of living individuals and 51 of deceased individuals	Bisulphite conversion	MAD = 5.35 years in training set based on living individuals<br>MAD = 4.98 years in test set of living individuals<br>MAD = 9.72 years in test set of deceased individuals
Correia Dias et al. [77]	Blood, bone, and teeth	185 samples from 65 healthy individuals for blood (23 males and 42 females – 1 to 94 years), 68 deceased individuals (53 males and 15 females – 24 to 91 years), 23 individuals for teeth (8 males and 15 females – 26 to 88 years), and 29 individuals for bone (25 males and 4 females – 26 to 81 years).	Bisulphite conversion	MAD = 6.06 years considering a multi-matrix model
Khan et al. [78]	Buccal swabs	26 samples from 26 individuals (13 males and 13 females – 1 to 65 years)	Bisulphite conversion	No significant correlations were found between methylation levels and the chronological age
DNA Methylation – Methylation Specific PCR (MSP)				
Cui et al. [79]	Blood	32 samples from 32 individuals (16 males and 16 females – 5 to 79 years)	Bisulphite conversion	4.29 < MAD < 5.95 years in training set (for the eighth CpG sites)<br>MAD = 4.35 years in the test set
Xin et al. [80]	Blood	90 samples from 90 individuals (46 males and 44 female)	Bisulphite conversion	MADs of approximately 6 years
Kondo et al. [81]	Teeth	29 samples from 29 individuals (14 males, 14	Bisulphite conversion	MAD of approximately 8.94 years

		females, and 1 unknown sex – 20 to 79 years)		
Ogata et al. [82]	Teeth	99 samples from 99 samples (57 males, 41 females, and 1 unknown sex – 20 to 85 years)	Bisulphite conversion	MAE = 6.69 years in training set<br>MAE = 8.28 years in test set
Soedarsono et al. [83]	Blood	43 samples from 43 individuals (28 males and 15 females – 11 to 20 years)	Bisulphite conversion	Strong correlation index between genes methylation levels and chronological age
DNA Methylation – Methylation sensitive high-resolution melting (MS-HRM)				
Oka et al. [84]	Saliva	113 samples from 113 individuals (54 males and 59 females – 0 to 75 years)	Bisulphite conversion	MAD = 11.1 years
Hamano et al. [85]	Blood	74 samples from 74 individuals (22 living individuals and 52 deceased individuals – 0 to 95 years)	Bisulphite conversion	MAD = 7.44 years
Hamano et al. [86]	Saliva	263 samples from 247 individuals (131 males and 116 females – 1 to 73 years) and 16 cigarette butts (29 to 51 years)	Bisulphite conversion	MAD = 5.96 years in training set based on saliva<br>MAD = 6.25 years in test set based on saliva<br>MAD = 5.64 years in test set based on cigarette buts
DNA Methylation – MassARRAY and microarray				
Giuliani et al. [87]	Teeth	22 samples (7 males and 15 females – 17 to 77 years)	Bisulphite conversion – EpiTYPER® system	MAD = 1.20 years
Yi et al. [88]	Blood	65 Samples from 65 individuals (40 males and 25 females – 11 to 72 years)	Bisulphite conversion - Sequenom MassARRAY®	Very high Pearson correlation indices, with values around an r of 0.91
Freire-Aradas et al. [90]	Blood	829 samples from 725 individuals (354 males and 371 females – 18 to 104 years) and 52 monozygotic female twin pairs (42 to 69 years)	Bisulphite conversion - EpiTYPER® system - Microarray for gene selection	MAD = 3.07 years based on blood MAD = 4.23 years based on samples from monozygotic twin pairs
Freire-Aradas et al. [91]	Blood	1330 samples from 209 individuals (2 to 18 years), 398 individuals (3 to 17 years), and 723 individuals (14 to 94 years)	Bisulphite conversion - EpiTYPER® system - Microarray for gene selection	MAE = 0.94 years in training set<br>MAE = 1.25 years in validation set
Freire-Aradas et al. [89]	Blood	1047 samples from 1047 individuals (477 males and 570 females – 2 to 104 years)	Bisulphite conversion - EpiTYPER® system	MAE = 3.36 years in training set using QRNN<br>MAE = 3.32 years in test set using QRNN<br>MAE = 3.41 years in training set using QRSVM<br>MAE = 3.45 years in test set using QRSVM
Zubakov et al. [59]	Blood	216 samples from 216 individuals (113 males and 103 females – 4 to 82 years)	Bisulphite conversion - EpiTYPER® system - Microarray for gene selection	MAD = 4.23 years analysing 43 methylation markers
Aanes et al. [92]	Blood	3289 samples from 973 individuals (12 to 25 years) and 2316 (1013 males and 1303 females - 10 to 60 years)	Bisulphite conversion - Microarray	MAD value from 0.7 to 1 year
Bocklandt et al. [93]	Saliva	128 samples from individuals (99 males and 29 females – 18 to 70 years)	Bisulphite conversion - Microarray	MAD = 5.2 years in combined model
DNA Methylation – Next Generation Sequencing (NGS)				

Zhang et al. [94]	Blood	1545 samples from 1191 healthy individuals and 354 rheumatoid arthritis patients	Bisulphite conversion – Illumina techniques	MAD = 3.90 years in test set of healthy subjects<br>MAD = 3.11 years in test set of affected individuals
Refn et al. [95]	Blood	128 samples from 64 individuals (32 males and 32 females, one for each age between 18 and 49 years)	Bisulphite conversion – Illumina techniques	Evaluated the stability of the markers over time
Vidaki et al. [9]	Blood, Saliva	2584 samples from 1156 healthy individuals (559 males and 597 females – 2 to 90 years), 106 female monozygotic twins, 1011 diseased individuals (434 males and 577 females – 17 to 91 years), and 46 individuals (11 to 76 years) for blood and 265 individuals for saliva	Bisulphite conversion - Illumina techniques	MAE = 3.3 years in training sets<br>MAE = 4.4 years in blind control test
Amiri Roudbar et al. [96]	Blood	4409 samples from 4409 individuals (10 to 101 years)	Bisulphite conversion - Illumina techniques	Significant and robust correlation indices
Hong et al. [97]	Saliva	280 samples from 280 individuals (171 males and 109 female – 18 to 73 years)	Bisulphite conversion – Illumina techniques	MAD = 3.83 years
Lau and Fung [98]	Blood	991 samples from 991 individuals (414 males and 577 females – 19 to 101 years)	Bisulphite conversion – Array Illumina	MAD = 3.74 years
Alsaleh and Haddrill [99]	Blood	754 samples from 754 individuals (0 to 88 years)	Bisulphite conversion - Illumina techniques	MAD = 4.5 years in training sets<br>MAD = 4.6 years in test set
Lee et al. [100]	Semen, Blood, and Vaginal fluid	163 samples from 157 males for semen (18 to 70 years) and 6 females for 3 blood and 3 vaginal fluid samples (21 to 56 years)	Bisulphite conversion – MPS	MAE ranging from 4 to 7 years based on different regression systems
Lee et al. [101]	Bone	66 samples from skeletal remains from 66 individuals (51 males and 12 females and 4 unknown sex – 31 to 96 years)	Bisulphite conversion - Illumina techniques	Moderate age correlation
Jung et al. [102]	Cartilage	85 samples from 85 deceased individuals (77 males and 8 females – 26 to 89 years)	Bisulphite conversion – MPS	MAE = 4.17 years in training sets<br>MAE = 4.97 years in test set
Hong et al. [103]	Saliva	322 samples from 322 individuals (161 males and 161 female – 18 to 65 years)	Bisulphite conversion - MPS	MAD = 23.42 years in test set
Hong et al. [104]	Blood	250 samples from 250 individuals (125 males and 125 female -20 to 74 years)	Bisulphite conversion - MPS	Strong correlation indices
Guan et all. [105]	Blood	90 samples from 90 individuals (48 males and 42 females – 0 to 91 years)	Bisulphite conversion – MPS	MAD = 5.23 years in training sets<br>MAD = 6.49 years in test set

Becker et al. [106]	Bone	190 samples from 190 cadavers (135 males and 55 female - 0 to 96 years)	Bisulphite conversion – MPS	MAE = 4.95 years
Woźniak et al. [107]	Blood, Buccal swabs, Bone	481 samples from 160 individuals for blood (80 males and 80 females – 1 to 75 years), 160 individuals for buccal swabs (80 males and 80 females – 2 to 80 years), and 161 individuals for bone (129 males and 32 females – 19 to 93 years).	Bisulphite conversion - MPS	MAE = 3.2 years based on blood<br>MAE = 3.7 years based on buccal cells<br>MAE = 3.4 years based on bone
Aliferi et al. [108]	Blood, Saliva, Semen	155 sample from 110 individuals for blood (11 to 93 years), 34 individuals for saliva (16 to 91 years), and 11 individuals for semen (23 to 50 years)	Bisulphite conversion - MPS	MAE = 4.1 years in blind test set
Naue et al. [109]	Blood	324 samples from 324 individuals (162 males and 162 females – 18 to 69 years)	Bisulphite conversion - MPS	MAD = 3.16 years in test set
Naue et al. [110]	Brain, bone, muscle, buccal swabs, and whole blood	144 samples, one for each biological material from 29 individuals except for one buccal swab for which PCR failure occurred (equal distribution of females and males – wide coverage of ages)	Bisulphite conversion - MPS	Considering the tissue-specific markers when different tissues are analysed
Ochana et al. [111]	Blood	> 300 samples from > 300 individuals (10 to 80 years)	Bisulphite conversion - Illumina techniques	MAE values between 1 and 3 years
Refn et al. [112]	Blood	148 samples from 148 individuals (74 males and 74 females – 18 to 68 years)	Bisulphite conversion - Illumina techniques	MAE = 2.58 years in training sets<br>MAE = 3.35 years in test set
Heidegger et al. [113]	Semen	2 samples from 2 males	Bisulphite conversion - MPS	MADs between 4.1 and 5.5 years
Pisarek et al. [114]	Semen	381 samples from 381 males (24 to 60 years)	Bisulphite conversion – MPS	MAE = 4.3 years based on 6CpG model in training set<br>MAE = 5.1 years based on 6CpG model in test set
Freire-Aradas et al. [115]	Cartilage	181 samples from 181 individuals (145 males and 36 females – 19 to 75 years)	Bisulphite conversion - MPS	MAE = 4.41 years in training set considering model 3<br>MAE = 4.26 years in test set considering model 3
Piniewska-Róg et al. [116]	Blood	212 samples from 212 deceased individuals (30 to 60 years)	Bisulphite conversion - Illumina techniques	MAE = 3.1 years in alcohol abusers<br>MAE = 3.3 years in control group
Pośpiech et al. [117]	Blood	5 samples from 5 individuals (7 to 78 years)	Bisulphite conversion – Ion Torrent and Illumina techniques	Smaller MAE considering Ion Torrent techniques = 2.7 years
Vidaki et al. [119]	Blood	1057 samples from 1057 males (15 to 87 years)	Bisulphite conversion - Illumina techniques	MAE = 7.54 years in validation set<br>MAE = 7.61 years in test set
Llobet [120]	Blood	617 samples from 617 individuals (285 males and 332 females)	Bisulphite conversion - Illumina techniques	Standard deviation error between 3.3 and 6.5 years
Mawlood et al. [121]	Blood	82 samples from 82 females (18 to 91 years)	Bisulphite conversion - Illumina techniques	MAD = 9.3 years
DNA Methylation – Single Base Extension (SBE) - SNaPshot				

Onofri et al. [122]	Blood	84 samples from 84 individuals (40 males and 44 females – 18 to 65 years)	Bisulphite conversion	MAD = 3.12 years in test set of healthy subjects<br>MAD = 3.01 years in test set of affected individuals
Grignani et al. [123]	Blood	101 samples from 72 living individuals (22 males and 37 females) and 29 burnt individuals (21 males and 8 females) – 18 to 85 years	Bisulphite conversion	MAD = 6.92 years based on carbonised individuals
Dias et al. [124]	Blood	121 samples from 59 living individuals (34 males and 38 females) and 62 cadavers (49 males and 13 females) – 1 to 94 years	Bisulphite conversion	MAD = 4.25 years based on living individuals<br>MAD = 5.36 years based on living individuals
Han et al. [125]	Blood	529 samples from 529 individuals (2 to 82 years)	Bisulphite conversion	MAE = 3.52 years using MLR model<br>MAE = 2.88 years using SVR model
Ye et al. [126]	Blood	145 samples from 145 individuals (11 to 71 years)	Bisulphite conversion	MAD = 4.73 years based on blood samples<br>MAD = 4.49 years based on fresh bloodstains<br>MAD = 5.43 years based on aged bloodstains
Filoglu et al. [127]	Blood	100 samples from 100 living individuals (60 males and 40 females – 20 to 83 years)	Bisulphite conversion	MAE = 3.75 years in sensitivity test<br>MAE = 4.07 years
Jiang et al. [128]	Blood	187 samples from 187 males (21 to 100 years)	Bisulphite conversion	MAD = 4.65 years using Random Forest model
Pan et al. [129]	Blood	310 samples from 310 individuals (189 males and 121 females – 2 to 86 years)	Bisulphite conversion	MAD = 4.22 years using stepwise regression model<br>MAD = 4.01 years using support vector model
Marcante et al. [130]	Saliva	60 samples from 60 individuals (30 males and 30 females – 23 to 70 years)	Bisulphite conversion	MAD = 3.49 years
Carlsen et al. [131]	Buccal swabs	230 samples from 230 individuals (102 males and 128 females – 1 to 88 years)	Bisulphite conversion	MAD = 4.68 years in training set<br>MAD = 4.70 years in test set
Takahashi et al. [132]	Buccal swabs	51 samples from 51 individuals (21 to 68 years)	Bisulphite conversion	MAD = 3.88 years using 10 ng DNA using locked nucleic acid (LNA) primers
Ambroa-Conde et al. [133]	Saliva and buccal cells	368 samples, one for each biological material from 184 individuals (21 to 86 years)	Bisulphite conversion	The combination of tissue-specific markers significantly improves prediction
Jung et al. [134]	Blood, saliva, and buccal swabs	448 samples from 304 individuals (18 to 74 years) 150 for blood, 150 for saliva, and 148 for buccal swabs.	Bisulphite conversion	MAD = 3.84 years for tissue-combined model
So and Lee. [135]	Blood and saliva	132 samples from 66 individuals for blood (34 males and 32 females – 18 to 65 years) and 66 individuals for saliva (33 males and 33 females – 18 to 69 years)	Bisulphite conversion	All analysed CpG sites displayed a strong correlation with age, independently from instrumentation
Hao et al. [136]	Hair	166 samples from different individuals (1 to 86 years)	Bisulphite conversion	MAD = 4.15 years in test set of 40 independent samples
Lee HY et al. [137]	Semen	94 samples from 94 males (20 to 73 years)	Bisulphite conversion	MAD = 4.2 years in training set<br>MAD = 5.4 years in test set

Lee JW et al. [138]	Semen	31 samples from 12 males for semen (24 to 57 years) and 19 from forensic evidences	Bisulphite conversion	MAD = 4.8 years considering semen from males<br>MAD = 5.2 years considering forensic evidences
Er et al. [139]	Semen	115 samples from 115 males (20 to 71 years)	Bisulphite conversion	MAE = 3.81 years considering all variables in a multivariate linear regression model
Xiao et al. [140]	Blood and semen	473 samples, 180 of which are one for each biological material from 90 males (22 to 51 years), 253 individuals for semen (22 to 67 years), and 40 individuals for blood (22 to 65 years)	Bisulphite conversion	MAE = 2.91 years in simplified model for semen<br>MAE = 2.59 years in final model for semen<br>MAE > 9.5 years for blood
Lee JE et al. [10]	Blood, saliva, and semen	24 samples for each of the twelve laboratories from different individuals (19 to 73 years)	Bisulphite conversion	MAE = 5.0 years for blood across all four experimental parts<br>MAE = 3.8 years for saliva across all four experimental parts<br>MAE = 2.7 for semen across all four experimental parts
So et al. [141]	Blood, saliva, and semen	458 samples, equally divided in gender, 197 from 197 individuals for blood (18 to 70 years), 199 from 199 individuals for saliva (18 to 70 years), and 62 from males for semen (19 to 55 years)	Bisulphite conversion	MAE = 3.78 years for blood in validation set<br>MAE = 3.56 years for saliva in validation set<br>MAE = 4.55 years for semen in validation set
DNA Methylation – Pyrosequencing				
Yang et al. [143]	Blood	241 samples from 241 individuals (128 males and 113 females – 10 to 79 years)	Bisulphite conversion	MAD = 2.80 years based on male samples<br>MAD = 2.93 years based on female samples
Fan et al. [144]	Blood	240 samples (1 to 81 years)	Bisulphite conversion	MAD = 1.29 years based on random forest regression
Park et al. [145]	Blood	765 samples from 765 individuals (385 males and 380 females from 10 to 90 years)	Bisulphite conversion	MAD = 3.16 years in training set<br>MAD = 3.35 years in validation set
Ji et al. [146]	Blood, saliva, and semen	97 samples from 87 individuals for blood (84 males and 3 females – 18 to 60 years), 5 males for saliva (20 to 24 years), and 5 males for semen (20 to 24 years)	Bisulphite conversion	MAD = 5.50 years in training set<br>MAD = 6.74 years in test set
Huang et al. [147]	Blood	135 sampled from 89 individuals (47 males and 42 females – 9 to 75 years), 40 individuals (11 to 70 years) and 6 individuals (12 to 65 years)	Bisulphite conversion	MAD = 7.87 years in combined dataset
Spólnicka et al. [148]	Blood	615 samples from 190 diseased individuals (76 males and 114 females – 12 to 76 years) and 425 healthy individuals (2 to 75 years)	Bisulphite conversion	MAE = 12.2 calculated for patients in early onset Alzheimer's disease patients<br>MAE = 5.7 assigned for age-matched healthy controls
Zbieć-Piekarska et al. [149]	Blood	427 sampled from 427 individuals (2 to 75 years)	Bisulphite conversion	MAD = 3.4 years in training set<br>MAD = 3.9 years in test set
Zbieć-Piekarska et al. [150]	Blood	427 sampled from 427 individuals (2 to 75 years)	Bisulphite conversion	MAD = 5.03 years in training set<br>MAD = 5.75 years in test set

Thong et al. [151]	Blood	333 sampled from 333 individuals (249 males and 84 females – 0 to 88 years)	Bisulphite conversion	MAD = 3.7 years for the artificial neural network model
Smeers et al. [152]	Blood	206 samples from 206 individuals (9 to 91 years)	Bisulphite conversion	MAD = 3.26 years for the quantile regression model
Feng et al. [153]	Blood	582 samples from 390 individuals (15 to 75 years) and 192 individuals (17 to 73 years)	Bisulphite conversion	MAD between 2.71 and 2.91 years in training set considering 9 CpG sites<br>MAD between 2.47 and 4.41 years in validation sets considering 9 CpG sites
Fleckhaus et al. [154]	Blood	204 samples from 102 individuals from Central Europe and 102 individuals from Middle East (in both cases one male and one female for all years between 18 to 68 years)	Bisulphite conversion	MAD = 2.72 years in Central Europe<br>MAD = 3.34 years in Middle East
Anaya et al. [155]	Blood	264 samples from 264 individuals (0 to 93 years)	Bisulphite conversion	MAD = 7.42 years in test set
Cho et al. [50]	Blood	100 samples from 100 individuals (50 males and 50 females – 20 to 74 years)	Bisulphite conversion	MAD = 4.18 years for Koreans
Xiao et al. [156]	Blood	350 samples from 42 individuals (21 males and 21 females – 18 to 62 years) and 308 individuals (167 males and 141 females – 0 to 86 years)	Bisulphite conversion	MAE = 2.79 year for male<br>MAE = 2.53 years for females<br>MAE = 3.33 years considering both sex
Kampmann et al. [157]	Blood	49 samples from 49 individuals (18 to 64 years)	Bisulphite conversion	MAD = 3.62 years
Sukawutthiya et al. [158]	Blood	136 samples from 100 living individuals (18 to 60 years) and 36 death individuals	Bisulphite conversion	MAD = 4.2 years obtained removing the early 20 years samples
Guan et al. [159]	Buccal swabs	461 samples (0 to 81 years)	Bisulphite conversion	MAD = 2.12 years in training set<br>MAD = 4.39 years in validation set
Shiga et al. [160]	Buccal swabs	102 samples from 102 individuals (79 males and 23 females – 21 to 77 years)	Bisulphite conversion	MAD = 3.88 years
Koop et al. [161]	Buccal swabs	215 samples from 73 cadavers (50 males and 23 females – 0 to 90 years) and 142 living individuals (58 males and 84 females – 0 to 89 years)	Bisulphite conversion	MAD = 7.8 years in validation set of living individuals<br>MAD = 9.1 years in validation set of cadavers
Mayer et al. [162]	Buccal mucosa	199 samples from 95 healthy individuals (44 males and 51 females – 0 and 18 years) and 104 diseased individuals (56 males and 48 females – 1 to 17 years)	Bisulphite conversion	MAE = 1.79 years in healthy individuals<br>MAE = 2.21 years in diseased individuals
Becker et al. [163]	Saliva	457 sampled from 368 German individuals (165 males and 203 females – 0 to 94 years) and 89 Japanese individuals (34 males and 55 females – 8 and 87 years)	Bisulphite conversion	MAE = 4.14 years for German individuals<br>MAE = 4.38 years for Japanese individuals
Siahaan et al. [164]	Buccal swabs	88 samples from 88 individuals (10 to 80 years)	Bisulphite conversion	MAE = 4.14 years considering all samples<br>MAE = 3.85 years considering individuals under 28 years
Pfeifer et al. [165]	Blood and buccal swabs	321 samples from 151 deceased individuals for blood (88 males and 63 females – 1 to 96 years), 149	Bisulphite conversion	MAD = 9.84 years for blood<br>MAD = 8.32 years for buccal swabs

		living individuals for buccal swabs (89 males and 60 females – 0 to 95 years), and 21 individuals for blood (20 to 65 years)		
Alghanim et al. [166]	Blood and saliva	163 samples from 72 individuals for blood (5 to 72 years) and 91 individuals for saliva (6 to 73 years)	Bisulphite conversion	MAD = 6.6 years in training set based on blood<br>MAD = 10.3 years in validation set based on blood<br>MAD = 5.8 years in training set considering single-locus model based on saliva<br>MAD = 8 years in validation set considering single-locus model based on saliva
Bekaert et al. [167]	Blood and teeth	235 samples from 169 deceased individuals for blood, 37 living individuals for blood (0 to 91 years), and 29 individuals for teeth (19 to 70 years)	Bisulphite conversion	MAD = 3.75 years based on blood<br>MAD = 4.86 based on teeth
Li et al. [168]	Semen, Blood samples, Vaginal fluid and buccal swabs	92 samples from 72 individuals, 55 sperm samples from males, 9 blood samples from 9 individuals, 4 vaginal secretions from females and 3 oral swabs and 21 vaginal swabs from a single volunteer (30 years).	Bisulphite conversion	MAD approximately of 4 years
Soares Bispo Santos Silva et al. [169]	Blood and Saliva	67 samples from 23 individuals for blood and 44 individuals for saliva (5 to 72 years)	Bisulphite conversion	MAD = 6.9 years based on GRIA2<br>MAD = 9.2 years based on NPTX2
Fokias et al. [170]	Nails	113 samples from 108 living individuals and 5 cadavers (equally divided into males and females – 0 to 96 years)	Bisulphite conversion	MAD = 4.76 years based on combined model in training set<br>MAD = 5.48 years based on combined model in test set
Fokias et al. [171]	Nails	91 samples from 86 living individuals (0 to 96 years) and 5 cadavers (29 to 68 years)	Bisulphite conversion	MAD = 5.61 years in test set
Weidner et al. [172]	Blood	151 samples from 151 indiduals (0 to 80 years)	Bisulphite conversion	MAD = 5.4 years in training set<br>MAD = 4.5 years in test set
Márquez-Ruiz et al. [173]	Teeth	65 samples form 65 individuals (15 to 85 years)	Bisulphite conversion	MAE = 5.08 years based on 9CpG model in from two genes
Zapico et al. [174]	Teeth	20 samples form 20 individuals (22 to 70 years)	Bisulphite conversion	MAE = 1.55 years
DNA Methylation – Other different techniques				
Yuen et al. [175]	Blood	10 samples form 10 individuals (5 males and 5 females – 25 to 76 years)	Bisulphite conversion – Nanopore sequencing	Correlation indices very strong (r = 0.86) compared to more traditional techniques
de Bruin et al. [176]	Blood	6 samples form 6 males (19 to 53 years)	Bisulphite conversion – Nanopore sequencing	ELOVL2 showed a very low Pearson's correlation coefficient
Noroozi et al. [177]	Blood and Buccal swabs	962 samples, 741 blood samples from 741 individuals (20 to 81 years) and 221 buccal swabs form	Bisulphite conversion – Microarray techniques	MAE = 2.47 years for blood samples using Skin&Blood model<br>MAE = 3.86 years for buccal swabs samples using Skin&Blood model

		221 individuals (20 to 81 years)		
Manco and Dias [178]	Blood	56 samples form 56 individuals (21 males and 35 females – 1 to 94 years)	Bisulphite conversion – droplet digital PCR	MAD approximately of 10 years
Dias and Manco [179]	Blood	58 samples form 58 individuals (16 males and 42 females – 1 to 93 years)	Bisulphite conversion – droplet digital PCR	MAD = 4.66 years final predictive model
Ho Lee et al. [180]	Saliva	76 samples from 76 individuals (23 to 58 years)	Bisulphite conversion – droplet digital PCR	MAD = 3.3 years final predictive model
Mawlood et al. [181]	Blood	80 samples form 80 females (18 to 91 years)	Enzymatic digestion– EpiTect qPCR assay	MAD = 7.2 years predictive model based on four genes
Li et al.[182]	Semen	282 samples from 282 males (22 to 67 years)	Bisulphite conversion – Double-Enzyme Reduced Representation Bisulfite Sequencing	MAE = 3.30 years
Qian et al. [183]	Blood	3312 samples form 3312 individuals (18 to 83 years)	Bisulphite conversion – Microarray techniques – Stepwise Conditional Epigenome-Wide Association Study	MAD = 3.20 years
DNA Methylation – Combined techniques				
Freire-Aradas et al. [184]	Blood	84 samples form 84 individuals (18 to 99 years)	Bisulphite conversion – EpiTYPER – Illumina techniques – Pyrosequencing – SNaPshot	MAE = 2.49 years using Illumina techniques<br>MAE = 4.71 years using SNaPshot<br>MAE = 3.14 years using EpiTYPER<br>MAE = 3.29 years using Pyrosequencing
Shi et al. [185]	Blood	124 samples from 124 individuals (78 males and 46 females – 6 to 15 years)	Bisulphite conversion – Microarray – X-ray – droplet digital PCR	MAE = 0.47 years in males and MAE = 0.34 years in females, combining the model of DNA methylation Age prediction with those of Dental Age and Skeletal Age
Xu et al. [186]	Blood	66 samples form 66 females (20 to 80 years)	Bisulphite conversion – Illumina techniques – Sequenom MassARRAY	MAD approximately of 2 years in the original dataset using support vector regression on 11 CpGs
Montesanto et al. [187]	Blood	330 samples from 330 individuals (146 males and 184 females – 20 to 100 years)	Bisulphite conversion – Sequenom MassARRAY – Pyrosequencing	MAE = 5.13 year in training set<br>MAE = 4.5 years in test set
Schwender et al. [188]	Buccal swabs	141 samples from 141 individuals (47 males and 94 females – 21 to 69 years)	Bisulphite conversion – Pyrosequencing – SNaPshot	MAD = 5.11 years using Pyrosequencing in training set<br>MAD = 5.33 years using Pyrosequencing in validation set<br>MAD = 5.16 years using SNaPshot in training set<br>MAD = 6.44 years using SNaPshot in validation set
